# Machine Learning–Guided Surface Strain Engineering in Connected Platinum–Nickel Nanoparticle Catalysts for Advanced Oxygen Reduction Performance

**DOI:** 10.1002/advs.76651

**Published:** 2026-07-20

**Authors:** Aparna Chitra Sudheer, Gopinathan M. Anilkumar, Hidenori Kuroki, Yuuki Sugawara, Takeo Yamaguchi

**Affiliations:** ^1^ Laboratory For Chemistry and Life Science Institute of Integrated Research Institute of Science Tokyo Yokohama Kanagawa Japan

**Keywords:** connected nanoparticles, machine Learning, oxygen reduction reaction, polymer electrolyte fuel cell, Pt‐skin, support‐free catalyst, surface strain engineering

## Abstract

Engineering the surface structure of catalysts is critical for achieving high intrinsic activity in the oxygen reduction reaction (ORR). We report a machine‐learning (ML)‐guided materials design strategy for the synthesis of support‐free, connected nanoparticle catalysts with enhanced activity. ML analysis of a dataset comprising 210 Pt‐based ORR catalysts quantitatively evaluated the relative importance of multiple structural, compositional, and electronic descriptors, identifying surface compressive strain (≈−4%) as an effective integrated descriptor strongly associated with ORR specific activity (SA). Guided by this insight, a H_2_‐annealing‐induced surface structuring approach was developed to construct an interconnected porous Pt–Ni nanoarchitecture with a Pt‐skin (≈3 atomic layers) and tunable compressive strain (≈−3%) over a Ni‐enriched subsurface. The optimized catalyst synthesized under 100% H_2_‐annealing exhibits exceptional ORR activity, with a SA of 5.1 ± 0.5 mA cm_Pt_^−^^2^, corresponding to a 12‐fold enhancement relative to commercial Pt/C. A linear correlation between surface strain and SA experimentally validates ML predictions and highlights the importance of strain‐engineered surfaces in electrocatalysis. Furthermore, the connected nanonetwork demonstrates remarkable electrochemical durability, retaining substantial compressive strain and exhibiting minimal Ni dissolution after 10,000 potential load cycles. This work establishes a generalizable materials design framework for developing next‐generation high‐activity, durable electrocatalysts for energy conversion technologies.

## Graphical abstract

Machine learning‐guided strain engineering enables highly active, durable, support‐free Pt–Ni nanonetwork catalysts for the oxygen reduction reaction. Analysis of a Pt‐based catalyst dataset identifies surface compressive strain as an effective descriptor associated with enhanced activity and provided practical design guidelines. Guided by these insights, a strain‐engineered interconnected Pt–Ni nanonetwork is developed through controlled H_2_ annealing, achieving twelve‐fold higher activity than commercial Pt/C with excellent durability.

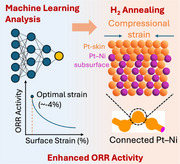

## 1 Introduction

Global energy demand and the urgent need to mitigate greenhouse gas emissions are driving the development of efficient and sustainable energy‐conversion technologies. Among the various emerging solutions, electrochemical devices that directly convert chemical fuels into electricity are particularly attractive due to their high efficiency and environmentally benign operation. Polymer electrolyte fuel cells (PEFCs), in particular, have attracted significant attention as next‐generation power sources for transportation and stationary energy applications. However, the practical implementation of PEFC technologies is fundamentally governed by the performance of electrode catalyst materials.

At the cathode, the oxygen reduction reaction (ORR) remains a critical kinetic bottleneck that limits overall device efficiency and increases precious metal requirements. Platinum‐based nanomaterials currently constitute the most effective ORR catalysts; nevertheless, their intrinsic activity remains substantially lower than the theoretical limits predicted by electrocatalytic principles [1]. Even the most advanced Pt‐based catalysts achieve specific activities (SAs) of approximately 10 mA cm_Pt_^−^^2^ [2, 3, 4], far below the theoretical maximum of ≈180 mA cm_Pt_^−^^2^ predicted by the ORR volcano plot for ideal plane surfaces [1]. Bridging this performance gap therefore requires precise control over catalyst surface atomic structure and electronic properties, highlighting the importance of rational materials design strategies.

Extensive research efforts have focused on engineering Pt‐based nanostructures with enhanced intrinsic activity. Previous studies have demonstrated that electrocatalytic performance can be tuned through a range of structural parameters, including ligand (ensemble) effects [5], lattice strain [6], surface and subsurface composition [7, 8], exposed crystal facets [9], coordination environment (step and edge density) [2], and microstructural features such as defects, grain boundaries [10], and microstrain [11, 12]. These structural factors collectively influence the Pt *d*‐band center and consequently alters the binding strength of oxygenated intermediates, thus dictating catalytic activity. For instance, low‐level Mo incorporation into Pt_3_Ni octahedra can markedly enhance ORR activity, yielding nearly an order of magnitude higher SA (≈10 mA cm_Pt_^−^^2^) than undoped Pt_3_Ni [4]. Theoretical and experimental studies suggest that Mo atoms preferentially occupy subsurface or edge sites, where they optimize oxygen‐binding energetics under operating conditions. In addition, the importance of the ligand effect has been demonstrated via the precise synthesis of Pd_3_Ru_1_/Pt core–shell nanoplates, that exhibit an exclusive ligand‐driven enhancement of ORR activity (≈7 mA cm_Pt_^−^^2^) [5]. Similarly, nanostructuring that generates undercoordinated surface atoms also promotes ORR kinetics; for example, ultrafine “jagged” Pt nanowires featuring abundant atomic steps and shortened Pt–Pt bonds demonstrated an SA (≈10 mA cm_Pt_^−^^2^) ≈33 times higher than that of Pt/C, attributable to the modified electronic structure at the highly strained, stepped surfaces [2]. Furthermore, controlled lattice compression, achieved through subsurface alloying [7, 8] or lanthanide incorporation [6], can generate compressively strained Pt‐skin overlayers that further enhance ORR activity ( ≈13 mA cm_Pt_^−^^2^) by weakening *OH/*O binding via downward d‐band shifts. In another study, strained Pt–Co core–shell nanoparticles supported on a Pt‐group‐metal (PGM)–free substrate exhibited remarkable ORR activity (≈10 mA cm_Pt_^−^^2^) with computational modeling revealing that interfacial electronic coupling between Pt–Co and PGM‐free sites optimizes binding energetics and stabilizes the active surface under operating conditions [3]. Despite these advances, the complex interplay among geometric and electronic effects makes it challenging to derive generalizable surface design principles using conventional experimental approaches alone.

Data‐driven approaches, particularly machine learning (ML), offer powerful opportunities to accelerate catalyst discovery by enabling rapid identification of key structure–activity descriptors from large experimental datasets [13, 14]. ML‐assisted analysis has already demonstrated considerable success in uncovering hidden correlations between material structure and catalytic performance in various electrochemical reactions, while accelerating the discovery of high‐performance nanocatalysts [15, 16, 17, 18, 19, 20]. For example, the authors’ previous ML‐assisted studies revealed the critical role of structural parameters in nonprecious metal oxides for oxygen evolution reaction activity [21, 22]. However, systematic ML‐guided design strategies aimed specifically at enhancing the intrinsic ORR activity of Pt‐based nanomaterials remain relatively underexplored.

Beyond intrinsic activity, catalyst durability under realistic fuel‐cell operating conditions represents another critical challenge for materials. Conventional carbon‐supported Pt nanoparticles are inherently vulnerable to severe degradation, suffering active surface area loss and catalyst detachment resulting from carbon corrosion under high‐potential operating conditions [23]. In recent years, advanced operational control systems have been implemented to suppress the severe potential excursions responsible for start–stop degradation. Consequently, the focus of research has shifted toward the challenge of load‐cycle durability, arising from repetitive potential cycling within a moderate voltage window (typically 0.6–0.95 V) during normal fuel cell operation. This process accelerates Pt dissolution and Ostwald ripening, catalyst nanoparticle aggregation, and the detachment of nanoparticles from the support, resulting in irreversible nanoparticle coarsening, reduced electrochemically active surface area, and gradual performance decay [24, 25]. To address these limitations, carbon‐free catalyst architectures based on interconnected Pt‐based nanoparticle networks have recently attracted increasing attention [26, 27, 28, 29]. Such support‐free nanoarchitectures form continuous conductive frameworks that can mitigate carbon‐related degradation while maintaining high catalytic accessibility and intrinsic activity. Membrane–electrode assemblies with carbon‐free cathode catalyst layers using the connected Pt_1_–Fe_1_ alloy catalyst retain 100% of their initial electrochemical surface area (ECSA) after 10,000 accelerated start–stop cycles [26]. Moreover, the connected Pt–Fe or Pt_3_–Co alloy with a chemically ordered structure shows high load cycle durability in an acidic electrolyte solution [28, 30]. These highly durable connected nanoparticles demonstrate high intrinsic activity, achieving ORR SAs of 1–3 mA cm_Pt_^−^^2^, approximately nine times higher than commercial Pt/C [26, 27, 28, 29].

**FIGURE 1:**
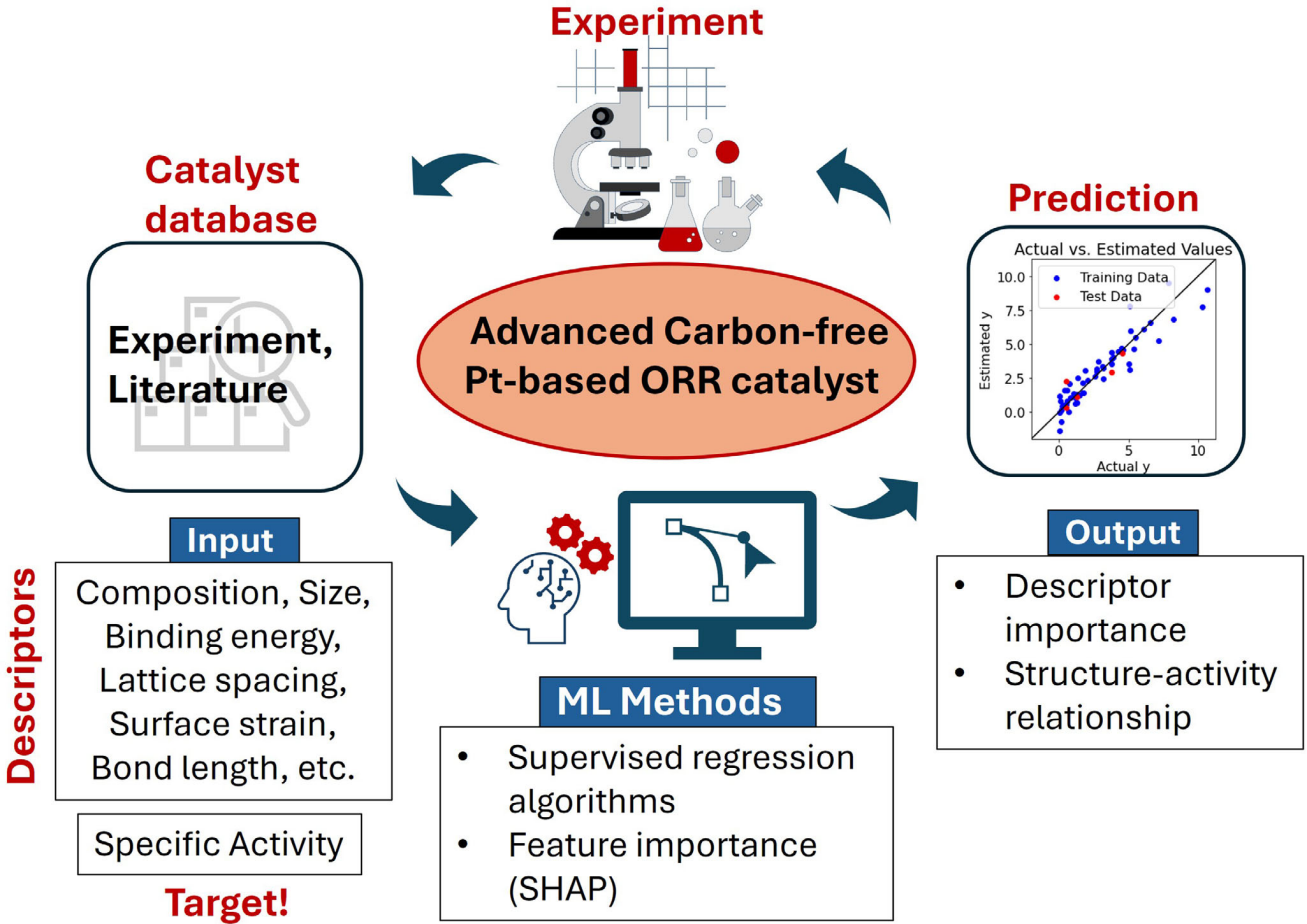
Schematic representation of the ML‐integrated workflow to develop advanced carbon‐free catalysts.

Building on these advances, the present study integrates machine learning‐guided catalyst design, literature‐derived datasets, and experimental catalyst development into a unified framework for advancing Pt‐based ORR catalysts. By identifying key descriptors governing ORR activity and applying atomic‐scale strain engineering within a unique interconnected carbon‐free nanostructure, we demonstrate a catalyst system that simultaneously achieves enhanced intrinsic activity and superior electrochemical durability. This combined data‐driven and materials‐engineering strategy, as illustrated in Figure 1, provides a generalizable framework for the rational design of next‐generation electrocatalyst materials for fuel‐cell energy‐conversion technologies.

## 2 Experimental Section

### 2.1 ML Framework

To establish a quantitative predictive model for ORR SA, a comprehensive dataset of 210 Pt‐based ORR electrocatalysts was compiled by integrating in‐house experimental data with selected literature reports. Table S1 lists ORR catalysts collected from the previously reported literature for the ML dataset. This dataset covered a broad range of catalyst types, including carbon‐supported (≈70%) and carbon‐free (≈30%) catalysts. It contained 24 descriptors capturing structural (e.g., *d*‐spacing, crystallite size, lattice parameter, surface strain), compositional (e.g., atomic%, alloying/dopant elements), and electronic properties (e.g., Pt 4*f*_7/2_ binding energy). While the beneficial effects of many of these descriptors on ORR activity have been widely reported, these factors were often mutually correlated, making it difficult to quantitatively assess their relative contributions through conventional experimental analysis alone. Therefore, the ML framework was employed to evaluate the relative importance of multiple interacting descriptors within a unified dataset and identify experimentally actionable design principles for high‐performance catalysts.

ML models were constructed using the scikit‐learn library (version 1.5.1) in Python (version 3.12.7) [31]. The ML processes were implemented using Spyder software (version 5.5.1) [32]. Before model training, missing values were replaced using mean imputation [33, 34], and all features were standardized through Z‐score normalization to maintain equal weighting. The dataset was divided into training and test sets using stratified sampling based on the SA distribution, resulting in an approximately 90:10 split. Specifically, 8 low‐SA samples (SA < 3), 5 mid‐SA samples (3 ≤ SA ≤ 7), and 3 high‐SA samples (SA > 7) were randomly selected for the test set (random state = 42), while the remaining samples were used for training. A series of supervised regression models was examined, including linear algorithms (Linear Regression, Multiple Linear Regression, Ridge, Lasso, Partial Least Squares (PLS)) and nonlinear algorithms (Random Forest Regressor, Extra Trees Regressor, Gradient Boosting Regressor (GBR), Support Vector Regressor). Model accuracy was evaluated using the coefficient of determination (R^2^), root mean squared error (RMSE), and mean absolute error (MAE) with cross‐validation applied to ensure statistical reliability. Hyperparameter optimization was performed using 5‐fold cross‐validation on the training set, and the list of hyperparameters used for different ML algorithms was shown in Table S2.

To gain mechanistic insight, SHapley Additive exPlanations (SHAP, version 0.47.2.) [35] analysis was employed. This approach enables simultaneous analysis of correlated factors and provides quantitative insight into their contributions to ORR activity.

### 2.2 Synthesis And Surface Tuning of Connected Nanoparticle Catalysts

**FIGURE 2:**
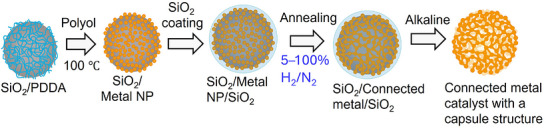
Schematic representation of the synthesis of connected nanoparticle catalysts.

Connected Pt–Ni nanoparticles were synthesized using a polyol‐assisted process, as illustrated in Figure 2. Platinum (II) acetylacetonate and nickel (II) acetylacetonate were dispersed in tetraethylene glycol with polyvinylpyrrolidone as a stabilizing agent. The precursor solution was then combined with PDDA‐functionalized SiO_2_ spheres and stirred for approximately 60 h to enable uniform adsorption of the metal precursors onto the template surface. Subsequently, Pt–Ni nanoparticles were formed by polyol reduction at 210°C for 2 h under a flowing Ar/H_2_ atmosphere, producing uniformly dispersed nanoparticles on the silica template. To preserve the connected nanostructure during subsequent thermal treatment, the composite was coated with a thin silica layer via a tetraethyl orthosilicate based sol–gel process at pH ≈ 10.5. The silica‐coated composite was then annealed at 500°C for 1 h under controlled H_2_/N_2_ atmospheres with 5, 50, and 100 vol% H_2_, to systematically investigate the influence of hydrogen concentration on structural evolution and ORR performance. Finally, both the silica template and the protective silica shell were removed by alkaline NaOH etching, yielding porous, capsule‐like connected Pt–Ni nanonetwork catalysts. Detailed reagent compositions, synthetic procedures, and experimental conditions were provided in the Experimental section of the Supporting Information.

### 2.3 Structural Characterization

The metal compositions of the catalysts were determined by inductively coupled plasma–atomic emission spectroscopy (ICP–AES). Crystal structures were characterized by X‐ray diffraction (XRD), from which crystallite sizes, and bulk lattice strain were determined. The morphology and nanostructure of the catalysts were examined by scanning electron microscopy (SEM), transmission electron microscopy (TEM), and high‐angle annular dark‐field scanning transmission electron microscopy (HAADF‐STEM). Surface strain was determined from the average Pt (1 1 1) interplanar spacing of the outermost atomic layers measured from HAADF‐STEM images, while elemental distributions and Pt‐shell thicknesses were analyzed by STEM–energy‐dispersive X‐ray spectroscopy (STEM–EDX). The electronic structures of the catalysts were investigated by X‐ray photoelectron spectroscopy (XPS). Detailed analysis methods, including the calculations of crystallite size, strain, Pt shell thickness, as well as instrument specifications, were provided in the Supporting Information.

### 2.4 Electrochemical Characterization

Electrochemical measurements were performed using a rotating disk electrode (RDE) system in 0.1 M HClO_4_. Catalyst inks were prepared by dispersing the catalysts in a 25% isopropanol solution containing Nafion (ionomer‐to‐catalyst ratio ≈ 0.05) and drop‐casting onto glassy carbon electrodes. ECSA was determined from the hydrogen desorption region of cyclic voltammograms (CV) recorded in N_2_‐saturated HClO_4_ at a scan rate of 20 mV s^−1^ after electrochemical pretreatment. ORR activities were evaluated by linear sweep voltammetry (LSV) in O_2_‐saturated HClO_4_ at room temperature using a rotation rate of 1600 rpm and a scan rate of 10 mV s^−1^. Catalyst durability was assessed by square‐wave potential cycling in N_2_‐saturated 0.1 M HClO_4_ at 60°C. Detailed instrument specifications, and analysis parameters were provided in the Supporting Information.

## 3 Results and Discussion

### 3.1 Evaluation Of ML Models and Descriptor Importance

**FIGURE 3:**
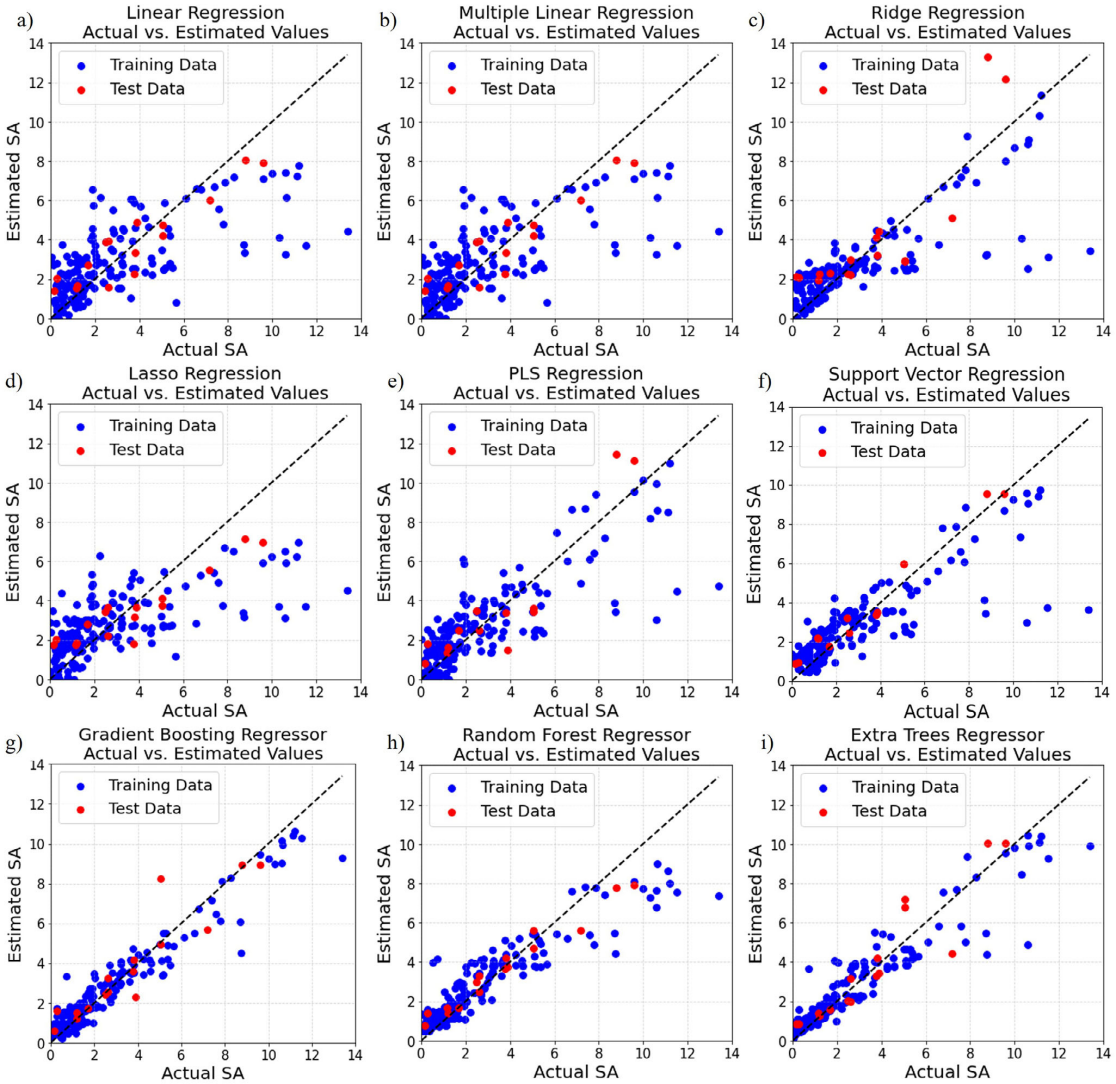
Comparison of actual versus predicted SA values for various ML regression models.

The performance of each ML model was evaluated by comparing the predicted SA values with the actual experimental results, as shown in Figure 3. As summarized in Table S3, for certain algorithms, prediction accuracy was notably higher at low‐to‐moderate SA values (below ≈6 mA cm_Pt_^−2^), where the majority of data points were concentrated. However, accuracy declined for high‐activity catalysts (SA > 7 mA cm_Pt_^−2^), likely because of the limited number of such data points and the increased complexity associated with their structure–activity relationships. Among all models tested, GBR demonstrated the highest overall performance, achieving an R^2^ of 0.92, RMSE of 0.80, and MAE of 0.54 on the training set, and an R^2^ of 0.85, RMSE of 1.06, and MAE of 0.66 on the test set. To assess the robustness of the highest‐performing GBR model across the full activity range, test‐set prediction errors were evaluated for separate SA bins; the resulting MAE and RMSE values are provided in Table S4.

**FIGURE 4:**
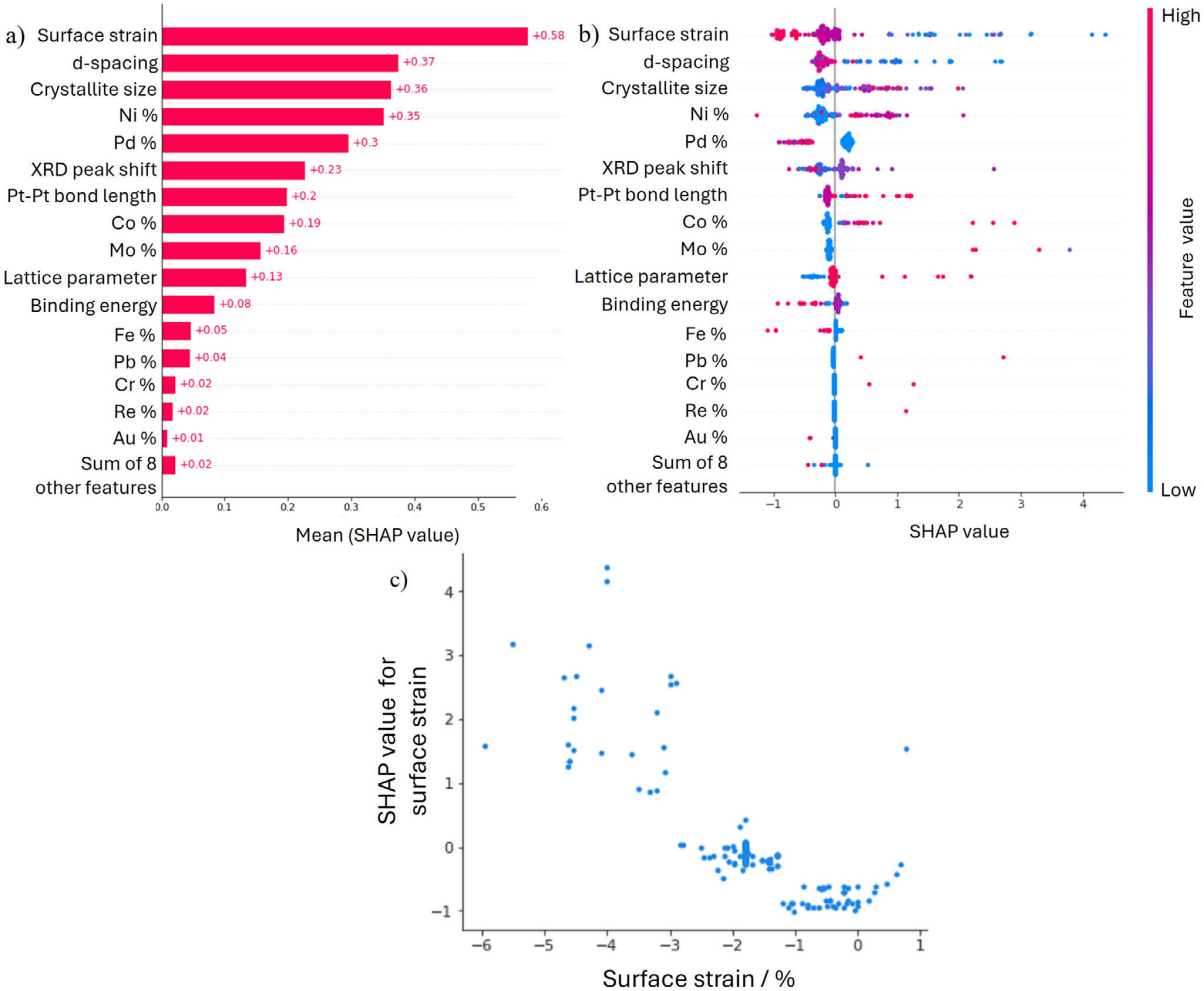
SHAP analysis results for the GBR model predicting SA of Pt‐based catalysts. (a) summary bar plot, (b) beeswarm plot, (c) dependence plot of surface strain versus SA.

The SHAP summary bar plot and beeswarm plot (Figure 4a,b) revealed that structural descriptors have a stronger impact on catalytic activity. Surface strain emerged as the most influential factor, followed by d‐spacing. Surface strain quantifies the localized lattice distortion at surfaces and is defined as the percentage deviation of the observed lattice spacing from the bulk Pt lattice spacing. In contrast, d‐spacing here represents the absolute distance between crystallographic planes (typically the (1 1 1) planes in face‐centered‐cubic (fcc) Pt alloys), directly measured from XRD peaks. While both descriptors reflect structural distortions, they capture complementary aspects: d‐spacing reflects bulk or average lattice parameters, whereas surface strain captures local distortions at surfaces, interfaces, and thin‐shell regions, often corroborated by high‐resolution TEM. Consequently, surface strain provides a more localized and chemically relevant metric, while d‐spacing provides an absolute structural measure, in combination providing a comprehensive understanding of catalytic behavior.

The SHAP dependence analysis (Figure 4c) further quantifies these effects. Higher SHAP values indicate a stronger positive contribution of a feature to the predicted ORR SA. For surface strain, lower (more compressive) values consistently correlate with increased SA, with the highest‐activity region predominantly distributed within a compressive strain range of approximately −3% to −5%. Within this favorable region, the model shows optimal predictions around ≈−4% compressive strain; however, this value should be interpreted as representing the center of a broader high‐activity trend rather than a sharply defined optimum. It should also be noted that several descriptors in the dataset are inherently correlated rather than fully independent. Therefore, the SHAP‐derived feature importance values should not be interpreted as completely independent contributions, but rather as a quantitative indication of which experimentally accessible descriptors most strongly correlate with ORR activity across the compiled dataset. The analysis suggests that surface strain acts as an effective integrated descriptor reflecting the combined influence of coupled structural and electronic modifications.

To the best of our knowledge, such systematic, data‐driven comparisons across Pt‐based ORR datasets remain limited. Therefore, this work represents an important initial step toward integrating machine learning with catalyst design and establishes a framework that can be extended to more complex electrocatalyst systems in future studies.

### 3.2 Structural Evolution of Connected Nanoparticle Catalysts

The ML analysis provided a practical synthesis/design direction for ORR catalyst development by highlighting strain control as a key parameter. While various strategies, such as defect‐rich surfaces, have been explored to induce lattice strain, these high‐energy sites are prone to dissolution or coalescence during electrochemical cycling [36]. To overcome these stability challenges, the formation of a stable Pt‐skin has emerged as a robust and widely adopted approach [37, 38]. In this study, thermal annealing was employed as a post‐synthetic surface‐tuning strategy to simultaneously introduce and control lattice strain and the Pt‐skin. H_2_‐annealed Pt–Ni [39, 40] and Pt–Co [8] systems can markedly improve activity by forming a compressively strained Pt‐skin over a transition‐metal‐rich subsurface. This configuration optimizes both geometric and electronic structures, facilitating oxygen intermediate adsorption and *OH desorption [41]. Here, ML analysis identified Ni as the most effective alloying element for enhancing Pt‐based ORR activity (Figure S1). Accordingly, Pt–Ni was selected as the base system for atomic‐scale tuning.

**FIGURE 5:**
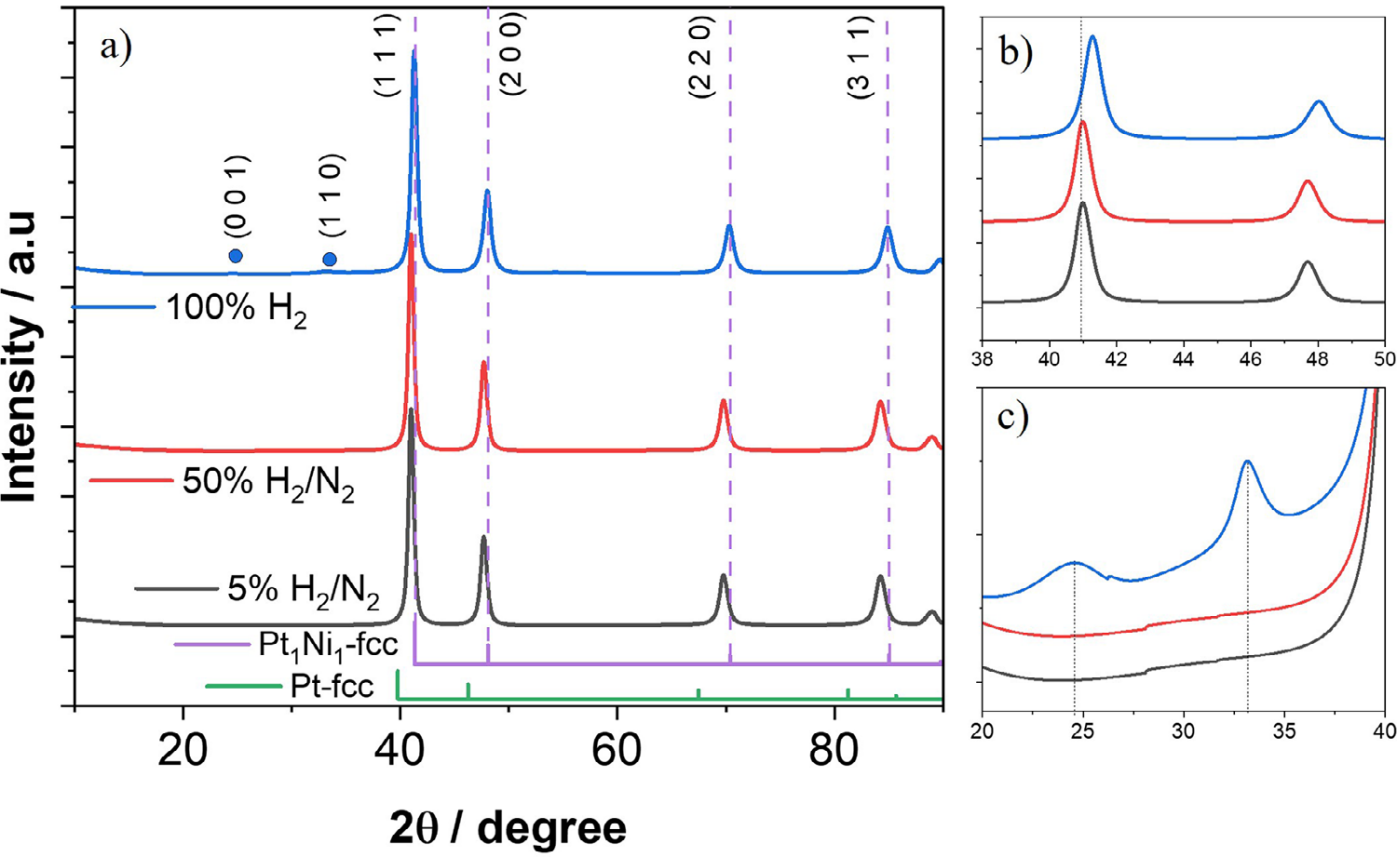
XRD patterns of Pt_1.5_–Ni catalysts annealed under varying H_2_ concentrations: 5%, 50%, and 100%. (a) full XRD profiles showing the fcc reflections (b) magnified view of the (1 1 1) peak shift toward higher 2θ values. (c) magnified view of the superlattice reflections for the catalyst annealed under 100% H_2_.

Compositional analysis by ICP‐AES confirmed that the Pt/Ni atomic ratio remained at approximately 1.5 for all samples annealed under different H_2_ concentrations (5%, 50%, and 100%). XRD was used to examine the structural evolution of the connected Pt_1.5_–Ni catalysts as a function of reducing atmosphere. As shown in the XRD patterns in Figure 5a, all samples exhibited the characteristic five reflections associated with the Pt fcc structure, (1 1 1), (2 0 0), (2 2 0), (3 1 1), and (2 2 2), indicating a predominantly single‐phase disordered fcc alloy. Standard references for fcc‐Pt (PDF #00‐004‐0802) and fcc‐Pt_1_Ni_1_ (PDF# 04‐003‐4660) are included for comparison. With an increase in H_2_ concentration, the (1 1 1) diffraction peak systematically shifted to higher 2θ values, from 40.89° (5% H_2_) to 40.95° (50% H_2_) and 41.24° (100% H_2_) (Figure 5b). Notably, the sample annealed under 100% H_2_ shows additional weak reflections near 25° and 34° corresponding to the (0 0 1) and (1 1 0) peaks (Figure 5c), indicative of the emergence of an ordered intermetallic PtNi phase [42, 43]. The crystallite size remained approximately 12 nm across all connected Pt_1.5_–Ni catalysts, suggesting that the observed structural changes arise from atomic rearrangements and surface ordering rather than bulk grain growth or sintering. The *d*‐spacing values of the Pt_1.5_–Ni catalysts were calculated using Bragg's law and decreased with increasing H_2_ concentration, from 2.218 Å (5% H_2_) to 2.203 Å (50% H_2_) and 2.187 Å (100% H_2_). This trend demonstrates that stronger reducing environments induce substantial lattice compression within the connected nanoparticle network. Using the d‐spacing of Pt (1 1 1) of pure Pt (2.266 Å) as a reference, the corresponding bulk‐average strain for each Pt–Ni catalyst was calculated to be −2.1% (5% H_2_), −2.8% (50% H_2_), and −3.44% (100% H_2_) from XRD‐derived d‐spacing [44], with the catalyst annealed under 100% H_2_ exhibiting the highest strain.

**FIGURE 6:**
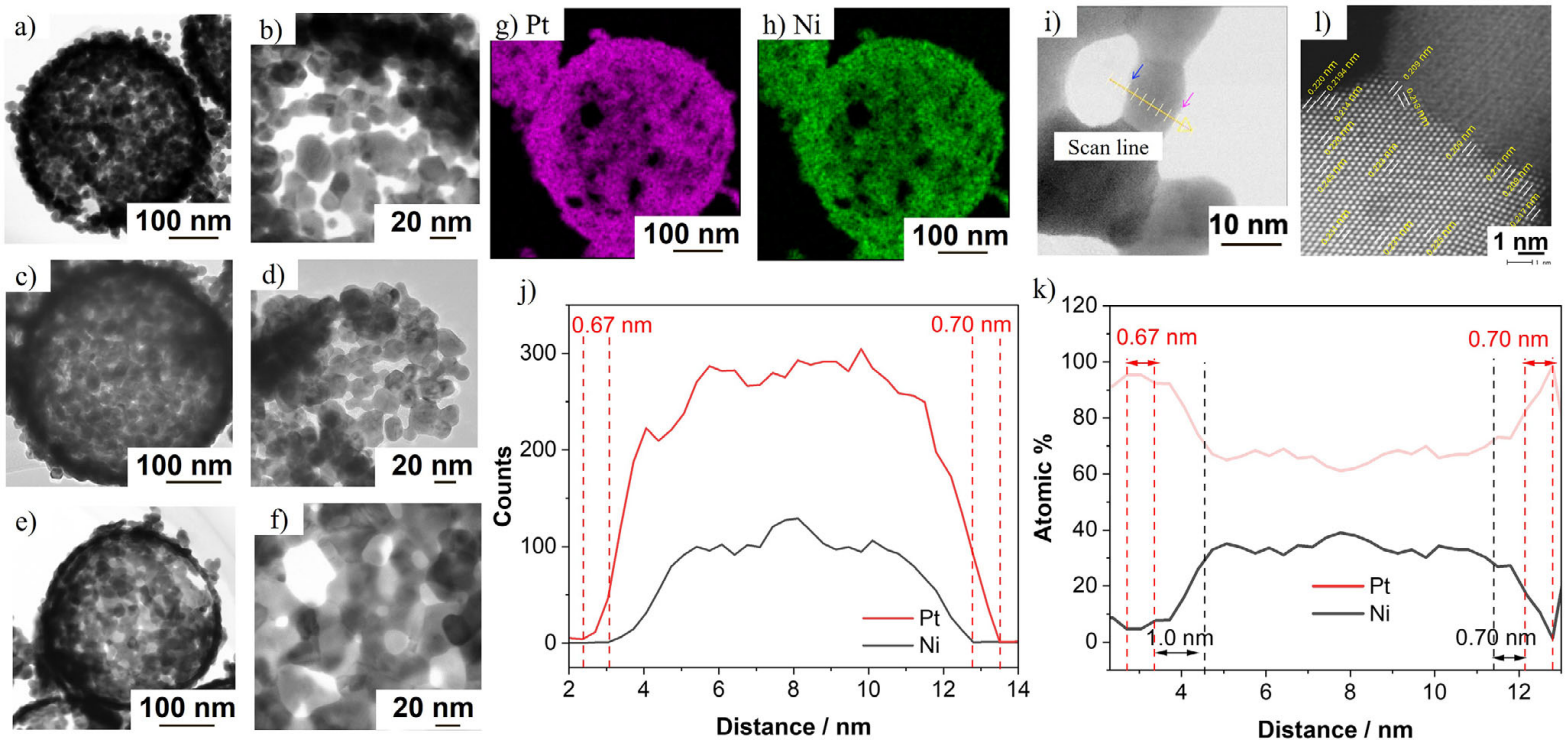
(a–f) STEM images of Pt_1.5_–Ni catalysts annealed at a, b) 5% H_2_, c, d) 50% H_2,_ and e, f) 100% H_2_. (g, h) EDX area scan, (i) STEM image, (j, k) corresponding EDX line scan profiles, and (l) STEM‐HAADF image of the Pt_1.5_–Ni catalyst annealed under 100% H_2_ atmosphere.

**FIGURE 7:**
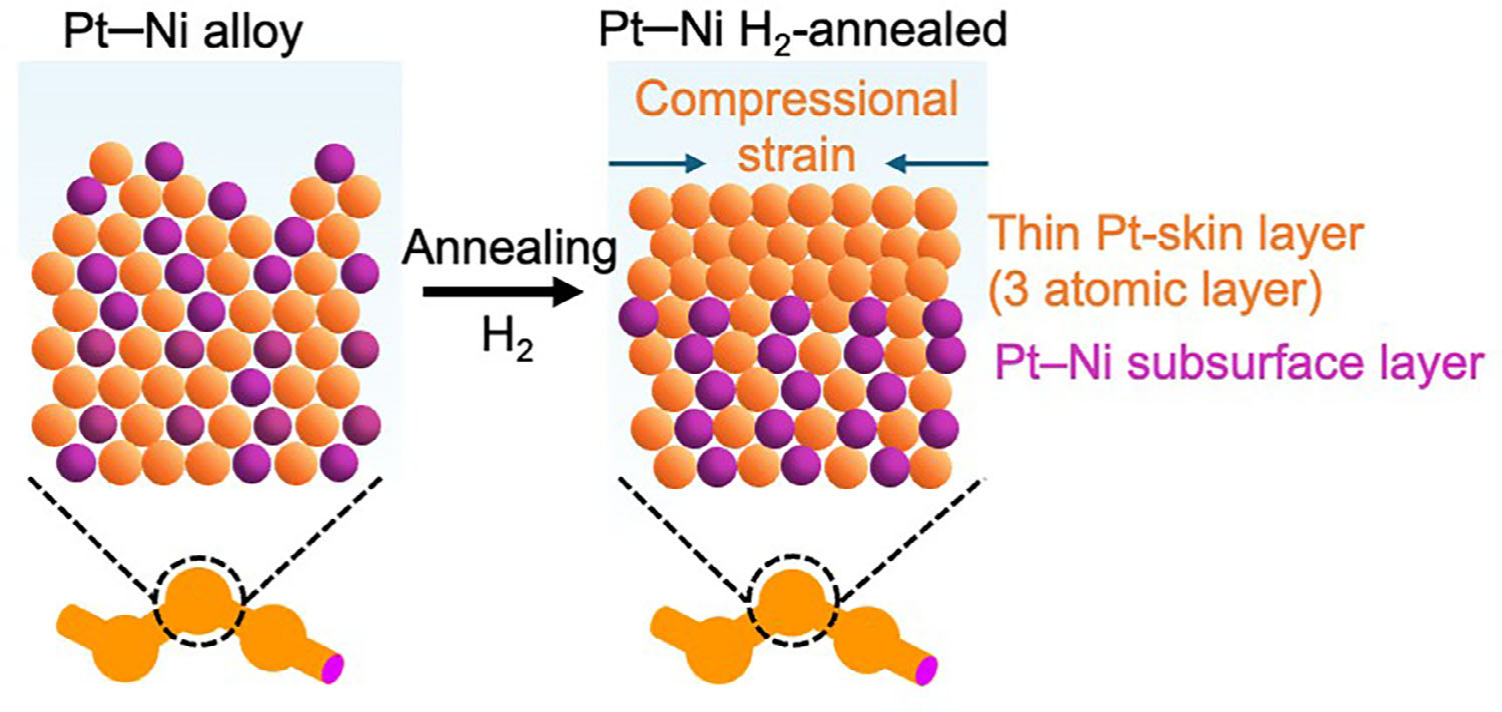
Schematic representation of thermal segregation during high‐temperature annealing under a hydrogen atmosphere.

Figure S2 shows SEM images of the synthesized catalysts showing porous, capsule‐like structure. The nanomorphology of the connected Pt_1.5_–Ni catalysts was examined using TEM. The TEM images of catalysts annealed under 5%, 50%, and 100% H_2_ (Figure 6a–f) reveal a highly interconnected nanoparticle network with well‐fused interfaces. The network thickness of the 100% H_2_‐annealed catalyst ranges from 8 nm to 18 nm, with an average of approximately 13 nm. EDX analysis was performed on the 100% H_2_‐annealed catalyst to investigate atomic elemental distribution. Area‐scan mapping (Figure 6g,h) confirms a uniform distribution of Pt and Ni. The STEM image and corresponding line‐scan profiles (Figure 6i–k and Figure S3a) reveal the formation of a thin Pt‐rich shell (0.69 ± 0.16 nm, n = 4; ≈3 atomic layers) and a Ni‐enriched subsurface region (37 ± 6% Ni within ≈1.0 ± 0.3 nm). Furthermore, HAADF‐STEM imaging of the 100% H_2_‐annealed catalyst (Figure 6l) displays distinct lattice fringes. The compressive strain within the Pt skin, calculated from the first ≈3 atomic layers based on line‐scan measurements, shows an average d‐spacing of 0.220 nm, corresponding to a surface strain of −3.05%. The bulk‐average strain, determined from an average of 15 measurements, was −3.35% (average d‐spacing 0.219 nm), consistent with the XRD‐derived bulk‐average strain. These results confirm the presence of a thin Pt atomic layer at the surface, underlain by a Ni‐rich Pt–Ni subsurface, which in combination generates enhanced compressive strain at the surface. The structural evolution observed in XRD patterns and internal lattice fringes can be attributed to the degree of alloying and the emergence of a superlattice‐type ordering within the Pt–Ni framework. These observations indicate that H_2_‐annealing promotes Pt surface segregation and Ni subsurface enrichment, as illustrated in Figure 7. During thermal annealing under H_2_, Pt, being more noble, segregates to the surface, while Ni diffuses into the subsurface to minimize surface energy. H_2_ facilitates this process by reducing surface oxides, particularly Ni oxides, creating a clean metallic surface that promotes controlled atomic rearrangement [45, 46, 47]. Thermodynamically, H_2_ lowers the surface free energy, favoring Pt enrichment, while kinetically accelerating the reduction of Ni^2+^ relative to Pt^2+^, driving Pt segregation toward the surface [48]. XPS analysis (Figure S4 and Table S5) confirms complete oxide removal for the 100% H_2_‐annealed catalyst, which exhibits only metallic Pt, whereas the 5% and 50% H_2_‐annealed catalysts retain minor PtO contributions.

### 3.3 ORR Activity of Connected Pt_1.5_–Ni Catalysts With Different Lattice Strain Levels

**FIGURE 8:**
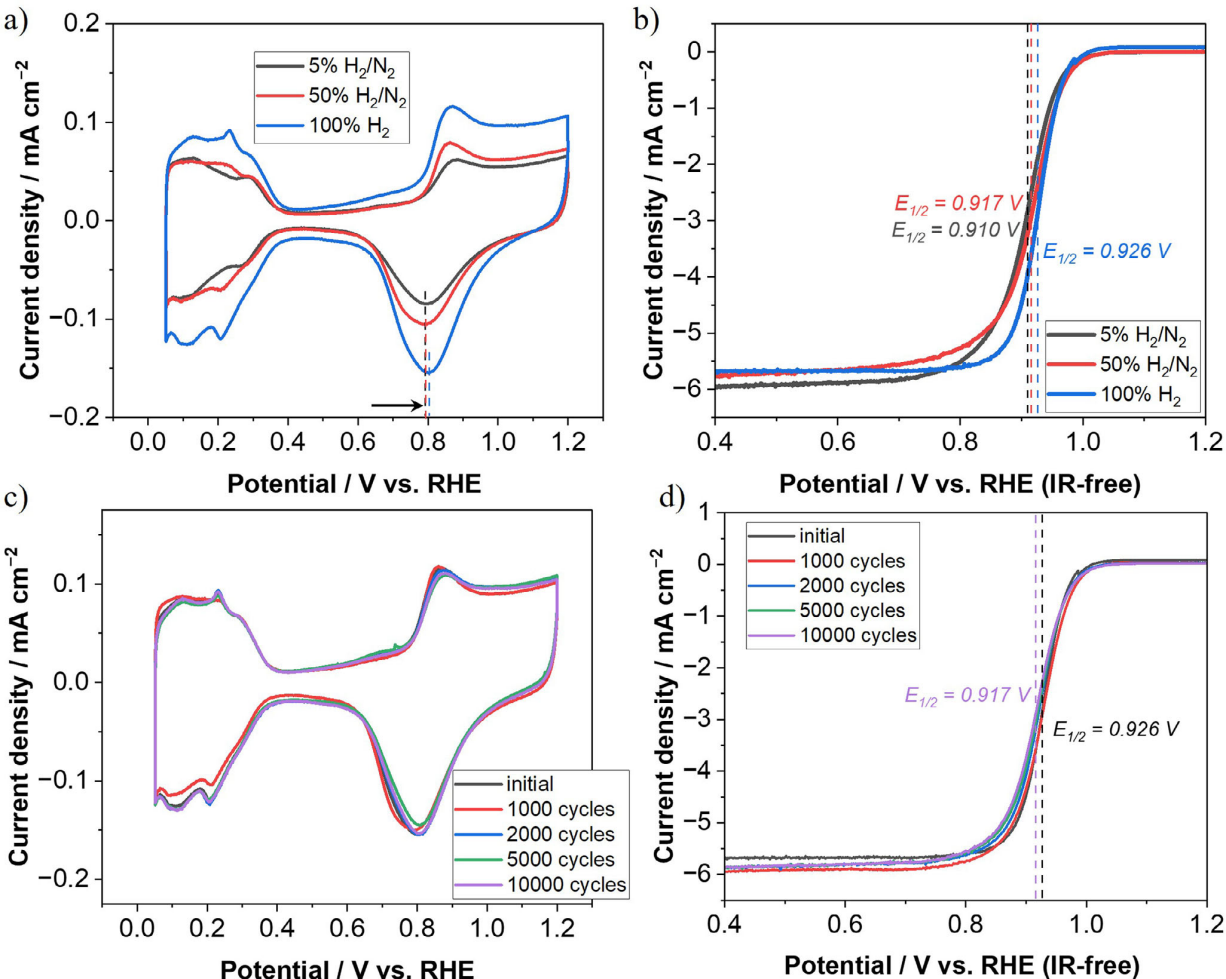
(a) CV curves and (b) LSV curves of Pt_1.5_–Ni catalysts annealed under 5%, 50%, and 100% H_2_ atmosphere. (c) CV curves and (d) LSV curves of Pt_1.5_–Ni catalysts annealed under 100% H_2_ atmosphere after different durability cycles.

To study the effect of H_2_‐annealing on catalytic performance, CV and LSV measurements were recorded for connected Pt_1.5_–Ni catalysts annealed under 5%, 50%, and 100% H_2_, as shown in Figure 8a,b. Surface reconstruction was evident from the hydrogen underpotential deposition (H_UPD_) region of the CV plot. The 5% and 50% H_2_‐treated catalyst exhibited a broad H_UPD_ feature corresponding to Pt (1 1 1), with a shoulder near 0.225 V vs. reversible hydrogen electrode (RHE), indicative of hydrogen adsorption on Pt (1 0 0). For the 100% H_2_‐annealed catalyst, this broad feature evolved into a double‐peak structure. The observed H_UPD_ evolution closely resembles that of mixed Pt (1 1 1)–(1 0 0) nanofacets [49], confirming that H_2_ annealing promotes reconstruction toward energetically favorable surface morphologies. The corresponding ECSA values were determined to be 18 ± 3, 19 ± 3, and 22 ± 2 m^2^ g_Pt_^−1^ for catalysts annealed under 5%, 50%, and 100% H_2_, respectively. In the oxide formation and reduction region (0.7–1.0 V vs. RHE), the *OH adsorption peak systematically shifted positively with increasing H_2_ concentration, from 0.789 V (5% H_2_) to 0.793 V (50% H_2_) and 0.800 V (100% H_2_), indicating a progressive weakening of *OH binding. This behavior reflects the formation of a compressively strained Pt‐rich skin with increased H_2_ annealing, as corroborated by the XRD and STEM–EDX analyses.

In the mixed kinetic–diffusion region of the LSV curve (0.80–0.95 V vs. RHE), higher H_2_ concentration for annealing improved ORR activity, with the half‐wave potential (E_1/2_) shifting positively from 0.910 V (5% H_2_) to 0.917 V (50% H_2_) and further to 0.926 V (100% H_2_), reflecting faster ORR kinetics and reduced overpotential. The mass activity (MA) and SA of the connected Pt_1.5_–Ni catalysts also increased progressively with H_2_ annealing, from 0.46 ± 0.02 A mg_Pt_^−^^1^ and 2.6 ± 0.3 mA cm_Pt_^−^^2^ for the 5% H_2_ sample, to 0.58 ± 0.05 A mg_Pt_^−^^1^ and 3.3 ± 0.4 mA cm_Pt_^−^^2^ for 50% H_2_, and reaching 1.13 ± 0.09 A mg_Pt_^−^^1^ and 5.1 ± 0.5 mA cm_Pt_^−^^2^ for the 100% H_2_‐annealed catalyst.

The enhanced ORR performance is associated with H_2_‐induced surface and subsurface reconstruction, as illustrated in Figure 7. The resulting Pt‐skin/Ni‐subsurface structure induces compressive strain in the Pt lattice, tuning the *d*‐band structure to weaken *OH/oxygen binding, thus promoting ORR kinetics [50]. The observations align with prior reports showing that strain engineering modulates the Pt *d*‐band and enhances ORR activity [2, 11, 51, 52, 53]. These structural and electronic changes are confirmed by XRD and STEM–EDX analyses of the 100% H_2_‐annealed catalyst, showing a thin Pt‐rich shell (0.69 ± 0.16 nm, ≈3 atomic layers) over a Ni‐enriched subsurface (37 ± 6% Ni within ≈1.0 ± 0.3 nm). The catalyst exhibited a surface strain of ≈−3% and bulk‐average strain of ≈−3.4%. These results suggest that the observed strain evolution is intrinsically linked to concurrent changes in Pt‐skin thickness and Ni surface/subsurface distribution induced by H_2_ annealing. Although higher H_2_ concentration promotes Pt surface segregation, the STEM–EDX results suggest that sufficient subsurface Ni remains preserved beneath the Pt‐rich shell, thereby enabling effective lattice contraction of the surface Pt layer. In addition, the partial formation of a chemically ordered structure under highly reducing conditions may further shorten subsurface interatomic distances and more efficiently transfer compressive strain to the outermost Pt surface. The relatively sharp hydrogen adsorption/desorption features observed in the CV profiles of the 100% H_2_‐annealed catalyst also suggest the formation of more ordered low‐defect Pt surface domains, which may further contribute to the enhanced ORR SA.

Overall, these results demonstrate that H_2_ annealing is an effective post‐synthetic strategy for tuning interconnected structural and electronic surface features, with strain serving as an effective descriptor of these coupled modifications. The connected Pt_1.5_–Ni catalyst annealed under 100% H_2_ exhibits exceptional ORR performance, achieving a MA of 1.13 ± 0.09 A mg_Pt_^−^^1^ and a SA of 5.1 ± 0.5 mA cm_Pt_^−^^2^. These values represent the highest reported to date among connected Pt‐based nanonetwork catalysts, surpassing systems such as Pt–Fe [26], Pt_3_–Co [28], and Pd@Pt [27]. In the context of carbon‐free catalyst architectures, the SA of the connected Pt–Ni system reported here is comparable to the top‐performing reported carbon‐free networks, such as self‐supported Pt–CoO (SA ≈5.38 mA cm_Pt_^−^^2^) [54] and Pt_3_Cu_97_ (SA ≈5.8 mA cm_Pt_^−^^2^) [55], while also exhibiting superior durability retention relative to these benchmarks. The connected Pt_1.5_–Ni catalyst shows approximately a 4‐fold increase in MA and a 12‐fold increase in SA compared with commercial Pt/C.

To elucidate the structure–activity relationship and validate the ML predictions, the compressive strain of the synthesized Pt_1.5_–Ni catalysts was plotted as a function of their SA. A clear linear correlation between surface compressive strain and SA was observed across different Pt‐based connected nanoparticle catalysts synthesized in this study and from previous studies (Figure S5) [26, 27, 28, 29]. Notably, the 100% H_2_‐annealed Pt_1.5_–Ni catalyst exhibited both the highest SA and the largest compressive strain (≈−3%), consistent with the ML‐predicted trend toward enhanced ORR activity with increasing compressive strain (Figure 3c). Although the experimentally achieved strain remains slightly below the ML‐predicted optimum (≈−4%), the results demonstrate that H_2_ annealing effectively enables strain tuning and substantial enhancement of ORR activity. Achieving higher compressive strain may require additional materials design or synthetic strategies beyond H_2_ concentration control, which remain an interesting direction for future investigation. Overall, the current experimental results provide compelling validation of the ML‐derived insight that compressive strain serves as an effective integrated descriptor associated with enhanced ORR activity, reflecting the coupled effects of lattice contraction, Pt‐skin formation, Ni‐enriched subsurface structure, and electronic modification.

### 3.4 Load Cycle Durability of 100% H_2_‐Annealed Connected Pt_1.5_–Ni Catalyst

Long‐term durability is essential to determine whether the induced compressive strain can be retained during extended operation, as prolonged cycling typically promotes leaching of transition metals and thickening of the Pt shell. To evaluate show that the H_UPD_ region remains nearly unchanged after 10,000 cycles, with the characteristic double peak associated with Pt (1 1 1)–(1 0 0) nanofacets still clearly visible. This indicates that the strain‐induced surface faceting and atomic configuration generated by H_2_‐annealing are well preserved. The ECSA decreased only slightly from 22 ± 2 to 20 ± 2 m^2^ g_Pt_^−^^2^, 1onfirming the retention of the hollow‐capsule nanonetwork architecture. Consistent with the CV results, the LSV patterns (Figure 8c,d) show only a modest decrease in kinetic current and a small negative shift in E_1/2_ from 0.926 V to 0.917 V, demonstrating excellent ORR stability. After 10,000 load cycles, the MA decreased to 0.67 ± 0.09 A mg_Pt_^−^^1^ and SA to 3.3 ± 0.5 mA cm_Pt_^−^^2^, corresponding to 60% MA and 65% SA retention. The majority of performance decay occurred within the first 2,000 cycles, after which activity stabilized, suggesting the surface reached a structural equilibrium. As illustrated in Figure S6, the evolution of ECSA, MA, and SA over cycling highlights the exceptional durability of the H_2_‐annealed Pt–Ni catalyst relative to commercial Pt/C. Even after 10,000 cycles, the catalyst retains superior activity, underscoring the robustness of its connected nanonetwork architecture.

A comparison of the ORR activity and load‐cycle durability performance of recently reported carbon‐free Pt‐based catalysts is provided in Table S6. When compared with catalysts evaluated under high‐temperature durability protocols (≥ 60°C), the catalyst developed in this study achieves a favorable combination of high ORR activity and excellent retention of both ECSA and ORR activity during load cycling.

**FIGURE 9:**
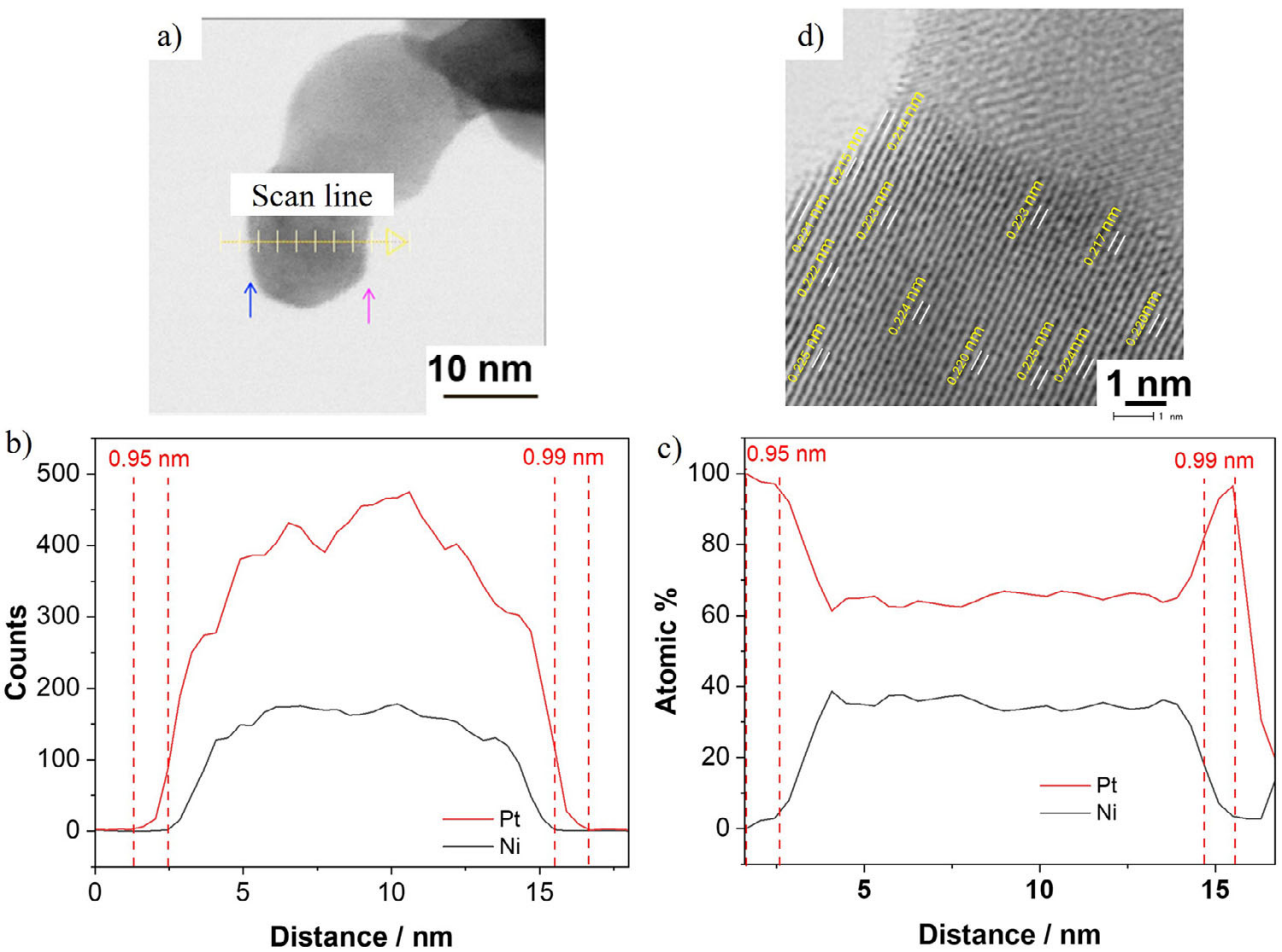
(a) STEM image, (b, c) corresponding EDX line scan profiles, and (d) HAADF‐STEM image of the Pt_1.5_–Ni catalyst treated under 100% H_2_ after durability test.

Results of post‐cycling structural characterization using STEM–EDX line mapping and HAADF‐STEM observation provided deeper insight into the atomic‐level durability of the catalyst (Figure 9). The EDX line profiles (Figure 9a–c and Figure S3b) confirm that the Pt‐skin structure remains intact after durability cycling, with the average Pt shell thickness increasing moderately from 0.69 ± 0.16 nm to 0.84 ± 0.16 nm after cycling, indicative of limited atomic diffusion and surface restructuring. Pt/Ni atomic ratio, calculated from the EDX results, changed only slightly from 1.92 before cycling to 1.75 after cycling, suggesting minimal compositional variation after durability testing. ICP analysis of the electrolyte solution after the durability test (10,000 load cycles) revealed a total metal loss of approximately 5% relative to the initial catalyst loading, indicating limited leaching under operating conditions. To further evaluate Ni dissolution, ICP analysis of the catalyst was performed. The bulk Pt/Ni atomic ratio showed only a small change from 1.5 before cycling to 1.7 after cycling, suggesting minimal Ni leaching during the durability test. These results confirm the excellent stability of the Pt‐skin layer, consistent with previous reports [37, 38]. These observations are further supported by previous in situ/operando studies on Pt‐based alloy catalysts that demonstrated Pt‐skin surface structures and alloy‐induced lattice contraction can remain stable under electrochemical operating conditions. Pt‐alloy nanoparticles with subsurface transition metals were reported to retain Pt‐skin/alloy‐core structures during potential cycling, while only limited near‐surface metal dissolution occurred [39, 56, 57]. The near‐surface lattice fringes (Figure 9d) indicate an average *d*‐spacing of 0.221 nm, corresponding to a surface strain of ≈−2.47%. This represents a slight relaxation from the pre‐cycling value of 0.220 nm (≈−3%) and confirms that the majority of the compressive strain was retained after long‐term durability testing. Therefore, the modest decrease in SA is primarily attributable to partial relaxation of compressive strain and slight Pt‐shell thickening, as verified by post‐cycling STEM–EDX analyses.

Overall, the strong preservation of catalytic activity and structural integrity is attributable to the robust, uniform Pt‐rich skin formed during H_2_ annealing, effectively suppressing Ni leaching. This engineered surface architecture produces a highly active and durable ORR interface, underscoring the critical role of controlled surface engineering, inducing favorable lattice strain for enhanced catalytic performance.

## 4 Conclusions

ML analysis was used to systematically integrate a broad dataset of experimentally reported Pt‐based catalysts and quantitatively identify practical design guidelines for high‐activity ORR catalysts. The analysis revealed that compressive strain acts as an effective integrated descriptor strongly correlated with ORR activity and suggested that highly active catalysts are generally located within a favorable compressive strain region centered near ≈−4%. Guided by these insights, we developed a new synthesis strategy based on controlled H_2_ annealing to tune the strain state of connected Pt–Ni nanoparticles, leading to the synthesis of novel carbon‐free Pt_1.5_–Ni connected nanoparticle catalyst. The optimized catalyst annealed under 100% H_2_ developed a Pt‐rich surface and a Ni‐enriched subsurface, achieving a high compressive surface strain of ≈−3%. This catalyst delivered an exceptional SA of 5.1 ± 0.5 mA cm_Pt_^−^^2^, and excellent durability, consistent with the ML‐predicted trend, highlighting the critical role of strain‐engineered surfaces in enhancing ORR performance. Notably, connected Pt–Ni nanonetworks are reported here for the first time, representing a new class of support‐free catalytic materials and demonstrating the successful translation of data‐driven catalyst design principles into experimental catalyst development.

The concepts demonstrated in this study, particularly the integration of ML, strain modulation and metal‐skin formation, are broadly applicable to other alloy systems and electrochemical reactions, providing a versatile framework for accelerating the rational design of next‐generation electrocatalysts.

## Author Contributions

**Aparna Chitra Sudheer**: conceptualization, data curation, formal analysis, investigation, methodology, software, visualization, Writing – original draft. **Gopinathan M. Anilkumar**: Writing – review and editing, supervision. **Hidenori Kuroki**: writing – review and editing, conceptualization, resources, supervision, validation. **Yuuki Sugawara**: writing – review and editing, software. **Takeo Yamaguchi**: conceptualization, funding acquisition, project administration, resources, supervision.

## Conflicts of Interest

The authors declare no conflicts of interest.

## Supporting information




**Supporting File**: advs76651‐sup‐0001‐SuppMat.docx.

## Data Availability

The data that support the findings of this study are available from the corresponding author upon reasonable request.
