# Supplementary material for: Machine Learning–Guided Surface Strain Engineering in Connected Platinum–Nickel Nanoparticle Catalysts for Advanced Oxygen Reduction Performance
Source: Adv Sci (Weinh). 2026 Jul 20;13(56):e76651. doi: 10.1002/advs.76651 (PMC13383700; doi:10.1002/advs.76651)
Supplement: Supplementary file 1 [file ADVS-13-e76651-s001.docx]

**Supporting Information**

**Machine Learning–Guided Surface Strain Engineering in Connected Platinum–Nickel Nanoparticle Catalysts for Advanced Oxygen Reduction Performance**

*Aparna Chitra Sudheer,^a^ Gopinathan M Anilkumar,^a^ Hidenori Kuroki,^a^* Yuuki Sugawara,^a^*

*Takeo Yamaguchi^a^**

^a^ Laboratory for Chemistry and Life Science, Institute of Integrated Research, Institute of Science Tokyo, Yokohama, Kanagawa 226-8501, Japan

*Corresponding authors, email: yamag@cls.iir.isct.ac.jp , kuroki@cls.iir.isct.ac.jp

# 1. **Experimental:**

1.1 Statistical Analysis

Table S1 List of ORR catalysts collected from previously reported literature for ML dataset.

| **Catalyst Name** | **Descriptor Availability (%)** | **Ref.** | **Catalyst Name** | **Descriptor Availability (%)** | | **Ref.** |
| --- | --- | --- | --- | --- | --- | --- |
| Pd@Cu@Pt/C | 91.7 | ^[1]^ | Fe_3_Pt/Ti_0.5_Cr_0.5_N; Fe_3_Pt/C | | 91.7 | ^[2]^ |
| PtFeBi \|; FePt \|; FePt@PtBi \| | 95.8 | ^[3]^ | (Pt_1.5_Ni; PtNi; PtNi_1.5_) | | 87.5 | ^[4]^ |
| PtP_2_@NPC | 95.8 | ^[5]^ | Pt/Pd/C | | 87.5 | ^[6]^ |
| Pd_49_Fe_21_@Pt_30_/C; Pd_17_Fe_31_@Pt_52_; Pd_46_Mn_6_@Pt_48_; Pd_15_Mn_5_@Pt_80_ | 95.8 | ^[7]^ | Pd_2_FeCo/C; Pd/C; PdFe/C PdCo/C | | 87.5 | ^[8]^ |
| Pd_9_Au_1_@Pt/C | 87.5 | ^[9]^ | L1_0_-CoPt@Pt | | 95.8 | ^[10]^ |
| PtPb@ Pt/C nanoplates; PtPb/C nanoparticles | 87.5 | ^[11]^ | Au_38.4_@Au_4.1_Pt_57.5_-NP/C; Au@AuPt NP/C | | 79.2 | ^[12]^ |
| Pd-Cu@Pt | 79.2 | ^[13]^ | PtNi/C | | 87.5 | ^[14]^ |
| PtCu/C; PtCuAu_0.0005_/C | 87.5 | ^[15]^ | PtNi(Mo)/C  Pt_3_Ni/C | | 83.3 | ^[16]^ |
| Pt_2.5_Ni/C | 87.5 | ^[17]^ | Pd@Pt-Ni/C | | 83.3 | ^[18]^ |
| Pt_3_Ni/C; M‐Pt_3_Ni /C (M: Mo, V, Cr, Mn, Fe, Co, W, Re) | 87.5 | ^[19]^ | A1-PtCo; L1_0_-PtCo; L1_0_-PtCo-M (M: W, Ga, Zn) | | 91.7 | ^[20]^ |
| PtFe@Gr/CB | 87.5 | ^[21]^ | O-Pt-Fe@NC/C | | 79.2 | ^[22]^ |
| Pd@Pt_monolayer_ nanosheets | 79.2 | ^[23]^ | PtCu_3_/C | | 79.2 | ^[24]^ |
| Pd-Pt-Ni (Octa1, Octa2, Octa3) | 79.2 | ^[25]^ | LP@PF-(1, 2) | | 100 | ^[26]^ |
| PtFe@NC/SWCNHs | 79.2 | ^[27]^ | PtFe@CS (6, 7, 8, 9)/C | | 79.2 | ^[28]^ |
| Pt_3_Mn intermetallic/C | 75.0 | ^[29]^ | PtBi/Pt core/shell nanoplates | | 87.5 | ^[30]^ |
| Pd@PtNi NSs (1, 2, 3) | 87.5 | ^[31]^ | Pt/Nb-SnO_2_ | | 79.2 | ^[32]^ |
| Nanocage PtNi/C | 79.2 | ^[33]^ | Nanoframed Pt_3_Ni/C | | 79.2 | ^[34]^ |
| Octahedral PtNi NPs/C | 83.3 | ^[35]^ | PtNi-BNCs/C | | 79.2 | ^[36]^ |
| Pd@Pt_nL_ NCs | 91.7 | ^[37]^ | PtNiCo NWs | | 79.2 | ^[38]^ |
| PtNWs , J-PtNWs, R-PtNWs | 87.5 | ^[39]^ | PtCu_3_Au_0.5_ NWP | | 79.2 | ^[40]^ |
| Pd@PtNi NWs | 79.2 | ^[41]^ | Pt*_x_*Y | | 83.3 | ^[42]^ |
| Pt_60_Fe_35_Mo_5_/C; Pt_35_Fe_60_Mo_5_/C; Pt_25_Fe_70_Mo_5_/C; Pt_60_Fe_35_V_5_/C; Pt_35_Fe_60_V_5_/C; Pt_25_Fe_70_V_5_/C; Pt_60_Fe_35_W_5_C; Pt_35_Fe_60_W_5_/C; Pt_25_Fe_70_W_5_/C Pt/C | 87.5 | ^[43]^ | PtNi/C PtNi/C Ni@Pt/C; hollow PtNi/C; sea sponge PtNi/C | | 83.3 | ^[44]^ |
| Pt_3_Ni/C dendrites  Pt_3_Ni/C dendrites | 79.2 | ^[45]^ | Pt_5_M (M: La, Ce, Sm, Gd, Tb, Dy, Tm, Ca) | | 83.3 | ^[46]^ |
| Hollow PtNi/C (25, 57, 80 ℃) | 83.3 | ^[47]^ | PtNi_3_ NPs | | 83.3 | ^[48]^ |
| PtNi_3_@OMC-A | 83.3 | ^[49]^ | Pt@Pt-skin Pt_3_Ni | | 79.2 | ^[50]^ |
| Pt–CoO (1, 2, 3, heat1, heat2) | 95.8 | ^[51]^ | Pt/p-BN | | 87.5 | ^[52]^ |
| Pt_3_Cu_97_ network | 87.5 | ^[53]^ | Connected Pt–Co | | 87.5 | ^[54]^ |
| Connected Pt–Fe | 87.5 | ^[55]^ | Connected Pd@Pt_0.3_, Pd@Pt_0.8_, Pd@Pt_1.5_ | | 91.7 | ^[56]^ |

The dataset comprises Pt-based ORR electrocatalysts collected from the literature. To improve data consistency, ORR activity values were selected, where available, from measurements performed under acidic conditions at or near room temperature, typically at 0.9 V vs. RHE, using a rotation speed of 1600 rpm, and iR-corrected polarization curves. All reported values were standardized to consistent units prior to analysis. Despite these efforts, slight variations in experimental conditions, including electrolyte composition and testing protocols among different literature sources, may contribute to inherent variability within the dataset.

Table S2 List of hyperparameters for ML algorithms^a^

| **Model** | **Hyperparameters** |
| --- | --- |
| a) Linear Regression (LR) | − |
| b) Multiple Linear Regression (MLR) | − |
| c) Ridge | 10^−6^ ≤ α ≤ 10^3^ |
| d) Lasso | 10^−6^ ≤ α ≤ 10^-1^ |
| e) Partial Least Squares (PLS) | n_components ∈ [1,2,3,…39] |
| f) Support Vector Regressor (SVR) | kernel = rbf, C ∈ [2^-3^, 2^0^, 2^3^, 2^7^], γ = 'scale',  ε ∈ [2^-7^, 2^-3^, 2^-1^, 2^0^] |
| g) Gradient Boosting Regressor (GBR) | n_estimators'∈ [200, 300], learning_rate∈ [0.05, 0.1, 0.2], max_depth∈ [3, 5, 7], min_samples_split∈ [2, 4] |
| h) Random Forest Regressor (RFR) | n_estimators ∈ [20, 50, 100], max_features ∈ [3, 5],  min_samples_split ∈ [2, 5, 10], max_depth ∈ [5, 10, 15],  min_samples_leaf ∈ [1, 2, 4] |
| i) Extra Trees Regressor (ETR) | n_estimators ∈ [100, 200], max_depth ∈ [None, 10, 20], min_samples_split ∈ [2, 5] |

^a^ The default values in each library were used for other nonmentioned hyperparameters.

## 1.2 Synthesis of Connected Pt_1.5_–Ni Nanoparticle Catalysts

***Precursor preparation:***
 Platinum(II) acetylacetonate (Pt(acac)_2_, Sigma-Aldrich), nickel(II) acetylacetonate (Ni(acac)_2_, Sigma-Aldrich), and polyvinylpyrrolidone (PVP, Mw ≈ 55,000 g mol^−1^, Sigma-Aldrich) were dissolved in tetraethylene glycol (TEG, Sigma-Aldrich). In a typical synthesis, 236 mg Pt(acac)_2_, 54.1 mg Ni(acac)_2_, and 2.2 g PVP were dispersed in 35 mL TEG under stirring.

***Template functionalization:***
 Silica spheres (average diameter: 320 nm; KE-P30, Nippon Shokubai Co., Ltd.) were coated with poly(diallyldimethylammonium chloride) (PDDA, Sigma-Aldrich) following the procedure used for connected Pt–Fe catalysts. ^[55,57]^ PDDA-modified SiO_2_ particles (35 mg in 25 mL TEG) were added to the Pt–Ni precursor solution and stirred for ~60 h.

***Polyol reduction:***
 Pt–Ni nanoparticles were deposited on the template by polyol reduction at 210 °C for 2 h in TEG under flowing Ar/H_2_, yielding uniformly distributed Pt–Ni nanoparticles on the SiO_2_/PDDA spheres. The product was cooled, washed with ethanol, and dried.

***Silica coating:***
 To inhibit nanoparticle agglomeration and detachment during annealing, the Pt–Ni/PDDA/ SiO_2_ composite was coated with a thin silica layer. The composite (120 mg) was dispersed in 3.48 mL DI water and 24 mL ethanol by sonication (5 min). Ammonium hydroxide (1.2 mL, 25%) and tetraethyl orthosilicate (0.6 mL, TEOS; Sigma-Aldrich) were added to maintain pH ≈ 10.5. The reaction proceeded for 6 h at room temperature under stirring. The silica-coated catalyst powder was washed with DI water and ethanol and dried at 60 °C overnight.

***Hydrogen annealing:***

The SiO_2_/Pt–Ni/PDDA/ SiO_2_ composite was annealed at 500 °C for 1 h in a tube furnace (ATF-DSP26B, ALPHA Engineering Inc., Japan or TMF-500N, AS ONE Corporation, Japan). Gas atmospheres with 5% H_2_/N_2,_ 50% H_2_/N_2_, and 100% H_2_ were used. A ramp rate of 10 °C min⁻^1^ ensured uniform heating.

***Template removal:***
 After annealing, both silica layers (template and coating) were removed by etching in 4 M sodium hydroxide (NaOH (aq.), Fujifilm Wako Pure Chemical Corporation) at 90 °C for 3 h. The sample was then washed thoroughly with DI water, revealing connected Pt–Ni nanonetworks with hollow, porous architecture.

## 1.3 Structural characterization

The metal compositions of the catalysts were determined by inductively coupled plasma-atomic emission spectroscopy (ICP‒AES; Shimadzu ICPS-8100). To investigate the crystal structures, X-ray diffraction (XRD) measurements were carried out using an X-ray diffractometer (Rigaku MiniFlex600C) operating at an accelerating voltage of 40 kV and a current of 15 mA using Cu Kα radiation (*λ* = 1.5406 Å). A Si non-reflective sample holder (Rigaku Corporation) was used, and the XRD data were collected over a 2*θ* range of 10−90° at a scan rate of 1° min^−1^. Reference patterns for fcc Pt (PDF #00-004-0802) and fcc Pt_1_Ni_1_ (PDF# 04-003-4660) were obtained from the International Center for Diffraction Data (ICDD). The crystallite sizes of the prepared catalysts were calculated using the Scherrer equation, which is expressed as *D* = *Kλ*/*β* cos *θ*, where *θ* is the Bragg angle, *β* is the full width at half maximum of the (1 1 1) peak, *λ* is the wavelength of the X-ray, and *K* is the shape factor (1.0747), assuming spherically shaped nanoparticles.^[58]^ Bragg's Law was used to find the d-spacing (distance between atomic planes in a crystal) using the formula *nλ*= 2*d* sin *θ* where *d* is the interplanar spacing, *n* is the order of reflection (an integer), *λ* is the wavelength of the X-rays, and 2*θ* is the diffraction angle, where *θ* is the angle of incidence. From the XRD-derived *d*-spacing (*d_sample,bulk_*) of the samples, the bulk-average strain was calculated as: bulk-average strain % = {(*d_sample,bulk_* – *d_Pt,bulk_*)/*d_Pt,bulk_*} × 100, where *d_Pt_* represent the distance between nearest fringes on the Pt (1 1 1) plane of pure Pt, estimated to be 2.266 Å.^[59]^

The morphology of the Pt_1.5_–Ni catalysts were analyzed by scanning electron microscopy (SEM, S-4800, Hitachi High-Technologies Corporation), and the nanoscale connected structures were examined using scanning transmission electron microscopy (STEM, JEM-ARM200F, JEOL) at an accelerating voltage of 200 kV. High-angle annular dark-field (HAADF) STEM imaging was used to resolve atomic-scale contrast. From these images, the average *d*-spacing of the Pt shell (*d_sample,surface_)* obtained from the first 3 near-surface atomic layers) was determined and used to calculate the surface strain as: surface strain % = {(*d_sample,surface_* – *d_Pt,bulk_*)/*d_Pt,bulk_*} × 100, (*d_Pt,bulk_ =* 2.266 Å).

Elemental distribution was further analyzed using energy-dispersive X-ray spectroscopy (EDX, JED-2300T, JEOL) mapping. The thickness of the Pt shell was estimated from the STEM–EDX line scan images. The Pt-shell boundary was defined from the STEM–EDX counts-versus-distance profile as the near-surface region where the Ni signal remained at background (negligible) levels while the Pt signal was dominant. The shell thickness was measured from the particle surface to the point at which the Ni counts began to increase consistently above the background level, indicating the transition to the Ni-enriched subsurface region. The electronic properties of the catalysts were examined using an X-ray photoelectron spectrometer (XPS, Quantera SXM, ULVAC-PHI Inc.) fitted with a twin-anode X-ray source using Al‒Kα radiation (hν = 1486.58 eV).

## 1.4 Electrochemical characterization

To make catalyst ink, the Pt_1.5_–Ni catalyst (Pt loading: 17.3 μg_Pt_ cm^−2^) was dispersed in a mixed low-Nafion solvent system containing 27 μL of 1-butanol (Fujifilm Wako Pure Chemical Corporation), 27 μL of 1-hexanol (FUJIFILM Wako Pure Chemical Corporation), 2.5 μL of 5 wt% Nafion solution (ionomer-to-catalyst ratio ≈ 0.05; Nafion 1100EW, Sigma–Aldrich Co. LLC), and 6.25 mL of a 25% isopropanol–deionized water solution (IPA, Fujifilm Wako Pure Chemical Corporation). The suspension was sonicated in an ice bath for over 1 hour to obtain a homogeneous catalyst ink. For electrochemical measurements, 10 μL of the ink (applied in two steps of 5 μL each) was drop-cast onto a glassy carbon disk electrode (geometric area: 0.196 cm^2^) and dried by rotating at 500 rpm under ambient conditions.

Electrochemical measurements were conducted using a potentiostat (HZ-7000, Meiden Hokuto Corporation) and an electrode rotating system (HZ-500, Meiden Denko Corporation) with a reversible hydrogen electrode (RHE) and a Pt wire as the reference and counter electrodes, respectively. The rotating disk electrode (RDE) method was used to evaluate the ORR and load-cycle durability of the connected Pt_1.5_–Ni catalysts. To stabilize the voltammograms, the catalyst electrodes were electrochemically pretreated by performing 50 cycles of cyclic voltammetry (CV) in the potential range of 0.05−1.2 V vs. RHE at a sweep rate of 50 mV s^−1^ in a N_2_-saturated 0.1 M HClO_4_ aqueous solution at room temperature. The ECSA was assessed by measuring the hydrogen desorption peaks in the CV curves of the electrochemically pretreated electrodes. Linear sweep voltammetry (LSV) measurements were performed in an O_2_-saturated 0.1 M HClO_4_ solution at room temperature with a rotation rate of 1600 rpm and a sweep rate of 10 mV s^−1^. The mass and specific ORR activities were determined by calculating the kinetic current at 0.9 V vs. RHE from the IR-corrected LSV curves and then dividing it by the mass and surface area of Pt on the catalyst electrodes. The load cycle durability of the catalysts was assessed by performing square-wave potential cycling in an N_2_-saturated 0.1 M HClO_4_ solution at 60 °C based on the Fuel Cell Commercialization Conference of Japan (FCCJ) protocol.^[60,61]^ The test involved potential cycling between 0.6 V and 0.95 V for 3 s each. The ECSAs and ORR activities were estimated after different load-cycle intervals. The stability of the catalyst structures was evaluated by TEM, STEM-EDX elemental mapping, and line-scan measurements after 10,000 load cycles. The above electrochemical tests were conducted under the same conditions using a commercial Pt/C catalyst (TEC10E50E, Tanaka Kikinzoku Kogyo K.K.) to obtain reference data.

# 2. **Results & Discussion:**

Table S3 Performance metrics of various supervised regression models used to predict the SA of Pt-based catalysts.

| **Model** | **R^2^  (Train)** | **R^2^  (Test)** | **RMSE**  **(Train)** | **RMSE**  **(Test)** | **MAE**  **(Train)** | **MAE**  **(Test)** |
| --- | --- | --- | --- | --- | --- | --- |
| a) Linear Regression (LR) | 0.47 | 0.86 | 2.00 | 1.07 | 1.41 | 0.97 |
| b) Multiple Linear Regression (MLR) | 0.48 | 0.86 | 1.99 | 1.07 | 1.39 | 0.97 |
| c) Ridge Regression | 0.60 | 0.59 | 1.74 | 1.84 | 1.07 | 1.46 |
| d) Lasso Regression | 0.51 | 0.10 | 1.52 | 2.00 | 1.0 | 1.21 |
| e) Partial Least Squares (PLS) | 0.65 | 0.77 | 1.63 | 1.38 | 1.07 | 1.14 |
| f) Support Vector Regressor (SVR) | 0.68 | 0.94 | 1.56 | 0.68 | 1.03 | 0.60 |
| g) Gradient Boosting Regressor (GBR) | 0.92 | 0.85 | 0.80 | 1.06 | 0.54 | 0.66 |
| h) Random Forest Regressor (RFR) | 0.72 | 0.89 | 1.46 | 0.95 | 0.95 | 0.77 |
| i) Extra Trees Regressor (ETR) | 0.88 | 0.90 | 0.96 | 0.92 | 0.62 | 0.71 |

Table S4 Test-set prediction performance (MAE and RMSE) as a function of SA bins

| **SA bin** | **No. of samples** | **MAE** | **RMSE** |
| --- | --- | --- | --- |
| Low (< 3) | 8 | 0.35 | 0.57 |
| Mid (3 ≤ SA ≤ 7) | 5 | 1.09 | 1.60 |
| High (> 7) | 3 | 0.76 | 0.95 |

ML analysis was conducted to evaluate the influence of various alloying metal elements on ORR activity. Several metals commonly reported in literature and from experimental studies, including Ni, Cu, Co, Mo, and others, were incorporated into the dataset. As shown in Figure S1, the Random Forest Regressor (RFR) model exhibited strong predictive capability, achieving an R^2^ of 0.71 together with low RMSE and MAE values. The accompanying SHAP summary bar plot further quantified the contribution of each alloying element to the model prediction, enabling clear identification of the metals most strongly associated with enhanced ORR activity.

Figure S1 Machine-learning results showing the influence of alloying metal elements on ORR activity. a) Parity plot comparing actual and predicted SA values obtained from the RFR, including the corresponding R^2^, RMSE, and MAE metrics. b) SHAP summary bar plot.

Figure S2 SEM images of connected Pt_1.5_–Ni catalysts annealed at a) 5% H_2_, b) 50% H_2_ and c) 100% H_2_

XPS was employed to investigate the surface chemical states and electronic environments of Pt and Ni in Pt_1.5_–Ni catalysts annealed under different hydrogen concentrations (5%, 50%, and 100%). As shown in Figure S3, the Pt 4*f*_7/2_ and 4*f*_5/2_ peaks for all the catalysts are centered around 71.45 eV and 74.25 eV, respectively. These values exhibit slight shifts (~+0.25 eV for Pt 4*f*_7/2_) compared to bulk metallic Pt (71.2 eV and 74.5 eV).^[62]^ This subtle shift is indicative of a modified electronic structure due to Pt–Ni alloying, where electron density around Pt atoms is influenced by the electronic interaction with Ni. Such behavior is consistent with d-band center modulation observed in alloyed systems, which can affect catalytic activity.^[63]^

To further elucidate the oxidation state of platinum, the Pt 4*f* spectra were deconvoluted into contributions from metallic Pt, Pt(II) (PtO), and Pt(IV) (PtO_2_) species as summarized in Table S2. The results show a trend of surface reduction with increasing H_2_ concentration. In the 100% H_2_-treated sample, only metallic Pt is detected, accounting for 34.3 atomic percent and 100% of the Pt 4*f* peak area, indicating complete removal of oxidized Pt species and the formation of a fully metallic Pt surface. In contrast, the 5% and 50% H_2_-treated catalysts exhibit small contributions from PtO (0.4–0.6 at%), which account for 1.6–2.0 % of the Pt peak area. These results confirm that lower hydrogen concentrations are insufficient for complete oxide reduction, whereas 100% H_2_ effectively eliminates surface Pt oxides. This effect of H_2_ annealing is previously reported in literatures.^[64,65]^

Figure S3 EDX line scan profiles of Pt_1.5_–Ni catalysts annealed at under 100% H_2_ atmosphere

a) before and b) after 10,000 durability cycles

Figure S4 XPS spectra of connected Pt_1.5_–Ni catalysts annealed under varying H_2_ concentrations: 5%, 50%, and 100%, in the Pt 4*f* region.

Table S5 XPS-derived quantification of Pt oxidation states in of connected Pt_1.5_–Ni catalysts annealed under varying H_2_ concentrations: 5%, 50%, and 100%.

| **Sample** | **Pt Atomic%** | | | **Pt Area%** | | |
| --- | --- | --- | --- | --- | --- | --- |
|  | **Metallic Pt** | **PtO** | **PtO_2_** | **Metallic Pt** | **PtO** | **PtO_2_** |
| **5% H_2_/N_2_** | 25.7 | 0.4 | 0.0 | 98.4 | 1.6 | 0.0 |
| **50% H_2_/N_2_** | 28.8 | 0.6 | 0.0 | 98.0 | 2.0 | 0.0 |
| **100% H_2_** | 34.3 | 0.0 | 0.0 | 100.0 | 0.0 | 0.0 |

Figure S5 SA as a function of compressive surface strain for the Pt-based connected nanoparticle catalysts obtained in this study and from previous studies.^[54,56,57,66]^

Figure S6 a) ECSA, b) MA, and c) SA of the connected Pt_1.5_–Ni catalyst and the commercial Pt/C catalyst before and after a specific number of load cycles. Error bars represent the standard deviation (SD) of three independent measurements (n = 3).

Table S6 Comparison of ORR activity and load cycle durability performances of recent carbon-free Pt-based catalysts using RDE measurements (@0.9 V vs. RHE) in acidic media.

| Catalyst/ Support | ECSA (m^2^ g_Pt_^−1^) | MA  (A mg_Pt_^−1^) | SA  (mA cm_Pt_^−2^) | Load Cycle Durability | | | | Ref. |
| --- | --- | --- | --- | --- | --- | --- | --- | --- |
|  |  |  |  | Conditions | ECSA  retention | MA retention | SA retention |  |
| Connected Pt_1.5_–Ni/ support-free | 22 ± 2 | 1.13 ±  0.09 | 5.1 ± 0.5 | 0.6–0.95 V,  60 °C,  10k cycles | 91% | 60% | 65% | This work |
| Connected Pd@Pt_0.8_/ support-free | 48 ± 3 | 0.59 ± 0.03 | 1.2 ± 0.2 | 0.6–1 V, 60 °C  10k cycles | 63% | 70% | 108% | ^[56]^ |
| Connected Pt_70_–Co_30_/ support-free | 8.1 | 0.23 | 2.84 | 0.6–1 V, 60 °C  10k cycles | 92% | 51% | 55% | ^[54]^ |
| Pt-CoO network/ support-free | 155.65  ± 4 | 8.37 ± 0.55 | 5.38 ± 0.21 | 0.6–1 V, 80 °C  3.6k cycles | 53% | 28% | 53% | ^[51]^ |
| Pt_3_Cu_97_ network/ support-free | 32 | 1.9 | 5.8 | 0.6–1 V, RT  10k cycles | 87% | 27% | 31% | ^[53]^ |
| PtPdIr nanospheres | 48.4 | 1.03 | 2.08 | 0.7–1.1 V, RT, 10k cycles | – | 78% | – | ^[67]^ |
| PtCu nanocrystals/  support-free | 100.39 | 1.125 | 1.120 | 0.6 and 1.1 V, 25 °C,  10k cycles | 93.0% | 89.8% | 97.2% | ^[68]^ |
| PtCu/Pr_0.15_Ce_0.85_O_2_ | 28.70 | 0.12 | 0.62 | 0.6–1 V, RT  30k cycles | – | – | 40% | ^[69]^ |
| Pt/Nb-SnO_2_ | 68* | 0.55 | 0.93 | 0.6–1 V, 80 °C  3k cycles | 88% | 87% | – | ^[32]^ |
| Pt/Ta-doped SnO_2_ | 73 ± 1 | 0.465 | 0.640 | 0.6–1 V, 20 °C  10k cycles | 83.6% | 76% | 90.6% | ^[70]^ |
| Pt/TiWC (2ML) | 60 | 0.420* | 0.690 | 0.4–1 V, RT  10.8k cycles | 91% | 78% | 86% | ^[71]^ |
| Pt NRs/GDC | – | 0.27 | 0.58* | 0.6–1 V, 80 °C  5k cycles | 89% | 87% | – | ^[72]^ |
| Pt/p-BN | 85.21 | 1.06 | 1.24 | 0.6–1.1 V, RT  10k cycles | 99.4% | 93% | – | ^[52]^ |

* Data are from the estimation of the data graph in the literature; RT: Room temperature,

NR: Nanorod, GDC: Gd-doped ceria, BN: Boron nitride.

**References**

[1]	D. Choi, J. Y. Jung, M. J. Lee, S. Kim, S. Lee, D. W. Lee, D. Kim, N. D. Kim, K.-S. Lee, P. Kim, S. J. Yoo, *ACS Catal.* **2021**, *11*, 15098.

[2]	Q. Liu, L. Du, G. Fu, Z. Cui, Y. Li, D. Dang, X. Gao, Q. Zheng, J. B. Goodenough, *Advanced Energy Materials* **2019**, *9*, 1803040.

[3]	J. Guan, S. Yang, T. Liu, Y. Yu, J. Niu, Z. Zhang, F. Wang, *Angew Chem Int Ed* **2021**, *60*, 21899.

[4]	C. Cui, L. Gan, M. Heggen, S. Rudi, P. Strasser, *Nature Mater* **2013**, *12*, 765.

[5]	W. Tian, Y. Wang, W. Fu, J. Su, H. Zhang, Y. Wang, *J. Mater. Chem. A* **2020**, *8*, 20463.

[6]	N. Aoki, H. Inoue, R. Yoshiura, Y. Hasegawa, S. Miyazaki, A. Suzuki, H. Daimon, T. Doi, M. Inaba, K. Higashi, T. Uruga, Y. Iwasawa, H. Tanida, Q. Yuan, N. Takao, H. Imai, T. Mikami, A. Daimaru, *J. Electrochem. Soc.* **2020**, *167*, 044513.

[7]	H.-U. Park, A.-H. Park, W. Shi, G.-G. Park, Y.-U. Kwon, *Ultrasonics Sonochemistry* **2019**, *58*, 104673.

[8]	W. Xiao, M. A. Liutheviciene Cordeiro, M. Gong, L. Han, J. Wang, C. Bian, J. Zhu, H. L. Xin, D. Wang, *J. Mater. Chem. A* **2017**, *5*, 9867.

[9]	J. Kong, Y.-H. Qin, T.-L. Wang, C.-W. Wang, *International Journal of Hydrogen Energy* **2020**, *45*, 27254.

[10]	J. Li, S. Sharma, X. Liu, Y.-T. Pan, J. S. Spendelow, M. Chi, Y. Jia, P. Zhang, D. A. Cullen, Z. Xi, H. Lin, Z. Yin, B. Shen, M. Muzzio, C. Yu, Y. S. Kim, A. A. Peterson, K. L. More, H. Zhu, S. Sun, *Joule* **2019**, *3*, 124.

[11]	L. Bu, N. Zhang, S. Guo, X. Zhang, J. Li, J. Yao, T. Wu, G. Lu, J.-Y. Ma, D. Su, X. Huang, *Science* **2016**, *354*, 1410.

[12]	X. Yue, X. Zhang, M. Zhang, W. Du, H. Xia, *Nanoscale* **2023**, *15*, 4378.

[13]	X. Li, Y. Liu, W. Bi, J. Bi, R. Guo, R. Li, C. Wang, Q. Zhan, W. Wang, S. Yang, F. Shi, J. Wu, M. Jin, *J. Mater. Chem. A* **2020**, *8*, 16477.

[14]	C. Cui, L. Gan, H.-H. Li, S.-H. Yu, M. Heggen, P. Strasser, *Nano Lett.* **2012**, *12*, 5885.

[15]	B.-A. Lu, T. Sheng, N. Tian, Z.-C. Zhang, C. Xiao, Z.-M. Cao, H.-B. Ma, Z.-Y. Zhou, S.-G. Sun, *Nano Energy* **2017**, *33*, 65.

[16]	F. Dionigi, C. C. Weber, M. Primbs, M. Gocyla, A. M. Bonastre, C. Spöri, H. Schmies, E. Hornberger, S. Kühl, J. Drnec, M. Heggen, J. Sharman, R. E. Dunin-Borkowski, P. Strasser, *Nano Lett.* **2019**, *19*, 6876.

[17]	S.-I. Choi, S. Xie, M. Shao, J. H. Odell, N. Lu, H.-C. Peng, L. Protsailo, S. Guerrero, J. Park, X. Xia, J. Wang, M. J. Kim, Y. Xia, *Nano Lett.* **2013**, *13*, 3420.

[18]	S.-I. Choi, M. Shao, N. Lu, A. Ruditskiy, H.-C. Peng, J. Park, S. Guerrero, J. Wang, M. J. Kim, Y. Xia, *ACS Nano* **2014**, *8*, 10363.

[19]	X. Huang, Z. Zhao, L. Cao, Y. Chen, E. Zhu, Z. Lin, M. Li, A. Yan, A. Zettl, Y. M. Wang, X. Duan, T. Mueller, Y. Huang, *Science* **2015**, *348*, 1230.

[20]	J. Liang, N. Li, Z. Zhao, L. Ma, X. Wang, S. Li, X. Liu, T. Wang, Y. Du, G. Lu, J. Han, Y. Huang, D. Su, Q. Li, *Angewandte Chemie International Edition* **2019**, *58*, 15471.

[21]	J.-H. Park, K. Kim, X. Wang, M. Huda, Y. Sawada, Y. Matsuo, N. Saito, M. Kawasumi, *Journal of Power Sources* **2023**, *580*, 233419.

[22]	Y. Hu, T. Shen, X. Zhao, J. Zhang, Y. Lu, J. Shen, S. Lu, Z. Tu, H. L. Xin, D. Wang, *Applied Catalysis B: Environmental* **2020**, *279*, 119370.

[23]	W. Wang, Y. Zhao, Y. Ding, *Nanoscale* **2015**, *7*, 11934.

[24]	M. Bele, P. Jovanovič, A. Pavlišič, B. Jozinović, M. Zorko, A. Rečnik, E. Chernyshova, S. Hočevar, N. Hodnik, M. Gaberšček, *Chem. Commun.* **2014**, *50*, 13124.

[25]	Y. Luo, W. Lou, H. Feng, Z. Liu, Q. Chen, G. Liao, X. Huang, P. Tsiakaras, P. Shen, *Catalysts* **2023**, *13*, 97.

[26]	L. Chong, J. Wen, J. Kubal, F. G. Sen, J. Zou, J. Greeley, M. Chan, H. Barkholtz, W. Ding, D.-J. Liu, *Science* **2018**, *362*, 1276.

[27]	S. He, Y. Liu, H. Zhan, L. Guan, *ACS Catal.* **2021**, *11*, 9355.

[28]	Y. Kim, H. E. Bae, D. Lee, J. Kim, E. Lee, S. Oh, J.-H. Jang, Y.-H. Cho, M. Karuppannan, Y.-E. Sung, T. Lim, O. J. Kwon, *Journal of Power Sources* **2022**, *533*, 231378.

[29]	J. Lim, C. Jung, D. Hong, J. Bak, J. Shin, M. Kim, D. Song, C. Lee, J. Lim, H. Lee, H. M. Lee, E. Cho, *J. Mater. Chem. A* **2022**, *10*, 7399.

[30]	Y. Qin, M. Luo, Y. Sun, C. Li, B. Huang, Y. Yang, Y. Li, L. Wang, S. Guo, *ACS Catal.* **2018**, *8*, 5581.

[31]	Q. Chen, Z. Chen, A. Ali, Y. Luo, H. Feng, Y. Luo, P. Tsiakaras, P. Kang Shen, *Chemical Engineering Journal* **2022**, *427*, 131565.

[32]	G. Shi, T. Tano, D. A. Tryk, A. Iiyama, M. Uchida, K. Kakinuma, *ACS Catal.* **2021**, *11*, 5222.

[33]	X. Peng, S. Zhao, T. J. Omasta, J. M. Roller, W. E. Mustain, *Applied Catalysis B: Environmental* **2017**, *203*, 927.

[34]	C. Chen, Y. Kang, Z. Huo, Z. Zhu, W. Huang, H. L. Xin, J. D. Snyder, D. Li, J. A. Herron, M. Mavrikakis, M. Chi, K. L. More, Y. Li, N. M. Markovic, G. A. Somorjai, P. Yang, V. R. Stamenkovic, *Science* **2014**, *343*, 1339.

[35]	R. Sakamoto, K. Omichi, T. Furuta, M. Ichikawa, *Journal of Power Sources* **2014**, *269*, 117.

[36]	X. Tian, X. Zhao, Y.-Q. Su, L. Wang, H. Wang, D. Dang, B. Chi, H. Liu, E. J. M. Hensen, X. W. (David) Lou, B. Y. Xia, *Science* **2019**, *366*, 850.

[37]	L. Zhang, L. T. Roling, X. Wang, M. Vara, M. Chi, J. Liu, S.-I. Choi, J. Park, J. A. Herron, Z. Xie, M. Mavrikakis, Y. Xia, *Science* **2015**, *349*, 412.

[38]	K. Jiang, D. Zhao, S. Guo, X. Zhang, X. Zhu, J. Guo, G. Lu, X. Huang, *Science Advances* **2017**, *3*, e1601705.

[39]	M. Li, Z. Zhao, T. Cheng, A. Fortunelli, C.-Y. Chen, R. Yu, Q. Zhang, L. Gu, B. V. Merinov, Z. Lin, E. Zhu, T. Yu, Q. Jia, J. Guo, L. Zhang, W. A. Goddard, Y. Huang, X. Duan, *Science* **2016**, *354*, 1414.

[40]	S. Zhang, H. Zhou, X. Liu, P. Tan, H. Liu, J. Pan, *Journal of Power Sources* **2023**, *567*, 232924.

[41]	Y. Zhao, L. Tao, W. Dang, L. Wang, M. Xia, B. Wang, M. Liu, F. Gao, J. Zhang, Y. Zhao, *Small* **2019**, *15*, 1900288.

[42]	P. Hernandez-Fernandez, F. Masini, D. N. McCarthy, C. E. Strebel, D. Friebel, D. Deiana, P. Malacrida, A. Nierhoff, A. Bodin, A. M. Wise, J. H. Nielsen, T. W. Hansen, A. Nilsson, I. E. L. Stephens, I. Chorkendorff, *Nature Chem* **2014**, *6*, 732.

[43]	J. Lu, L. Luo, S. Yin, S. W. Hasan, P. Tsiakaras, *ACS Sustainable Chem. Eng.* **2019**, *7*, 16209.

[44]	R. Chattot, T. Asset, P. Bordet, J. Drnec, L. Dubau, F. Maillard, *ACS Catal.* **2017**, *7*, 398.

[45]	X. Zhao, S. Takao, K. Higashi, T. Kaneko, G. Samjeskè, O. Sekizawa, T. Sakata, Y. Yoshida, T. Uruga, Y. Iwasawa, *ACS Catal.* **2017**, *7*, 4642.

[46]	M. Escudero-Escribano, P. Malacrida, M. H. Hansen, U. G. Vej-Hansen, A. Velázquez-Palenzuela, V. Tripkovic, J. Schiøtz, J. Rossmeisl, I. E. L. Stephens, I. Chorkendorff, *Science* **2016**, *352*, 73.

[47]	L. Dubau, M. Lopez-Haro, J. Durst, F. Maillard, *Catalysis Today* **2016**, *262*, 146.

[48]	L. Gan, S. Rudi, C. Cui, M. Heggen, P. Strasser, *Small* **2016**, *12*, 3189.

[49]	K. Wang, Y. Wang, S. Geng, Y. Wang, S. Song, *Advanced Functional Materials* **2022**, *32*, 2113399.

[50]	H. Jin, Z. Xu, Z.-Y. Hu, Z. Yin, Z. Wang, Z. Deng, P. Wei, S. Feng, S. Dong, J. Liu, S. Luo, Z. Qiu, L. Zhou, L. Mai, B.-L. Su, D. Zhao, Y. Liu, *Nat Commun* **2023**, *14*, 1518.

[51]	G. W. Sievers, A. W. Jensen, J. Quinson, A. Zana, F. Bizzotto, M. Oezaslan, A. Dworzak, J. J. K. Kirkensgaard, T. E. L. Smitshuysen, S. Kadkhodazadeh, M. Juelsholt, K. M. Ø. Jensen, K. Anklam, H. Wan, J. Schäfer, K. Čépe, M. Escudero-Escribano, J. Rossmeisl, A. Quade, V. Brüser, M. Arenz, *Nat Mater* **2021**, *20*, 208.

[52]	Q. Li, L. Li, X. Yu, X. Wu, Z. Xie, X. Wang, Z. Lu, X. Zhang, Y. Huang, X. Yang, *Chemical Engineering Journal* **2020**, *399*, 125827.

[53]	G. W. Sievers, J. R. Bowen, V. Brüser, M. Arenz, *Journal of Power Sources* **2019**, *413*, 432.

[54]	Q. Liao, H. Kuroki, T. Tamaki, M. Arao, M. Matsumoto, H. Imai, T. Yamaguchi, *ACS Appl. Nano Mater.* **2025**, *8*, 3323.

[55]	T. Tamaki, H. Kuroki, S. Ogura, T. Fuchigami, Y. Kitamoto, T. Yamaguchi, *Energy Environ. Sci.* **2015**, *8*, 3545.

[56]	A. C. Sudheer, G. M. Anilkumar, H. Kuroki, T. Yamaguchi, *Advanced Science* **2025**, *12*, 2408614.

[57]	H. Kuroki, T. Tamaki, T. Yamaguchi, *J. Electrochem. Soc.* **2016**, *163*, F927.

[58]	B. Akbari, M. Pirhadi Tavandashti, M. Zandrahimi, *Iranian Journal of Materials Science and Engineering* **2011**, *8*, 48.

[59]	Y. Zhang, J. Qin, D. Leng, Q. Liu, X. Xu, B. Yang, F. Yin, *Journal of Power Sources* **2021**, *485*, 229340.

[60]	A. Ohma, K. Shinohara, A. Iiyama, T. Yoshida, A. Daimaru, *ECS Trans.* **2011**, *41*, 775.

[61]	Y. Hashimasa, T. Shimizu, Y. Matsuda, D. Imamura, M. Akai, *ECS Trans.* **2013**, *50*, 723.

[62]	J. C. Fuggle, N. Mårtensson, *Journal of Electron Spectroscopy and Related Phenomena* **1980**, *21*, 275.

[63]	J. Greeley, I. E. L. Stephens, A. S. Bondarenko, T. P. Johansson, H. A. Hansen, T. F. Jaramillo, J. Rossmeisl, I. Chorkendorff, J. K. Nørskov, *Nat Chem* **2009**, *1*, 552.

[64]	G. A. Attard, J.-Y. Ye, A. Brew, D. Morgan, P. Bergstrom-Mann, S.-G. Sun, *Journal of Electroanalytical Chemistry* **2014**, *716*, 106.

[65]	S. Polani, K. E. MacArthur, J. Kang, M. Klingenhof, X. Wang, T. Möller, R. Amitrano, R. Chattot, M. Heggen, R. E. Dunin-Borkowski, P. Strasser, *ACS Appl. Mater. Interfaces* **2022**, *14*, 29690.

[66]	T. Tamaki, H. Kuroki, S. Ogura, T. Fuchigami, Y. Kitamoto, T. Yamaguchi, *Energy Environ. Sci.* **2015**, *8*, 3545.

[67]	K. Deng, Y. Xu, Z. Dai, H. Yu, S. Yin, Z. Wang, X. Li, L. Wang, H. Wang, *Chemistry An Asian Journal* **2019**, *14*, 3868.

[68]	X. Tan, J. Luo, J. Huang, K. Chen, P. Chen, H. Wu, H. Zhai, P. Tan, J. Pan, *Journal of Solid State Chemistry* **2026**, *361*, 126089.

[69]	T. Zou, Y. Wang, F. Xu, *ACS Appl. Mater. Interfaces* **2023**, *15*, 58296.

[70]	I. Jiménez-Morales, F. Haidar, S. Cavaliere, D. Jones, J. Rozière, *ACS Catal.* **2020**, *10*, 10399.

[71]	D. Göhl, A. Garg, P. Paciok, K. J. J. Mayrhofer, M. Heggen, Y. Shao-Horn, R. E. Dunin-Borkowski, Y. Román-Leshkov, M. Ledendecker, *Nat. Mater.* **2020**, *19*, 287.

[72]	G. Shi, T. Tano, D. A. Tryk, A. Iiyama, M. Uchida, Y. Kuwauchi, A. Masuda, K. Kakinuma, *Journal of Catalysis* **2022**, *407*, 300.
